# Life-long control of cytomegalovirus (CMV) by T resident memory cells in the adipose tissue results in inflammation and hyperglycemia

**DOI:** 10.1371/journal.ppat.1007890

**Published:** 2019-06-20

**Authors:** Nico A. Contreras, Katarzyna M. Sitnik, Ilija Jeftic, Christopher Patrick Coplen, Luka Čičin-Šain, Janko Nikolich-Žugich

**Affiliations:** 1 Department of Immunobiology and the University of Arizona Center on Aging, University of Arizona College of Medicine–Tucson, Tucson, AZ, United States of America; 2 Research Group Immune Aging and Chronic Infections, Department of Vaccinology, Helmholtz Centre for Infection Research, Braunschweig, Germany; 3 Department of Pathophysiology, Faculty of Medical Sciences, University of Kragujevac, Kragujevac, Serbia; 4 Cluster of Excellence RESIST (EXC 2155), Hannover Medical School (MHH), Hannover, Germany; Thomas Jefferson University, UNITED STATES

## Abstract

Cytomegalovirus (CMV) is a ubiquitous herpesvirus infecting most of the world’s population. CMV has been rigorously investigated for its impact on lifelong immunity and potential complications arising from lifelong infection. A rigorous adaptive immune response mounts during progression of CMV infection from acute to latent states. CD8 T cells, in large part, drive this response and have very clearly been demonstrated to take up residence in the salivary gland and lungs of infected mice during latency. However, the role of tissue resident CD8 T cells as an ongoing defense mechanism against CMV has not been studied in other anatomical locations. Therefore, we sought to identify additional locations of anti-CMV T cell residency and the physiological consequences of such a response. Through RT-qPCR we found that mouse CMV (mCMV) infected the visceral adipose tissue and that this resulted in an expansion of leukocytes in situ. We further found, through flow cytometry, that adipose tissue became enriched in cytotoxic CD8 T cells that are specific for mCMV antigens from day 7 post infection through the lifespan of an infected animal (> 450 days post infection) and that carry markers of tissue residence. Furthermore, we found that inflammatory cytokines are elevated alongside the expansion of CD8 T cells. Finally, we show a correlation between the inflammatory state of adipose tissue in response to mCMV infection and the development of hyperglycemia in mice. Overall, this study identifies adipose tissue as a location of viral infection leading to a sustained and lifelong adaptive immune response mediated by CD8 T cells that correlates with hyperglycemia. These data potentially provide a mechanistic link between metabolic syndrome and chronic infection.

## Author summary

Mouse cytomegalovirus (mCMV) infection results in initial systemic viremia that is thereafter controlled by the adaptive immune system. Control is mediated in part by T cells that render the virus undetectable systemically, and latent in specific organs, including the lungs and salivary glands. It remains unclear how latent virus is controlled across tissues given the large pool of systemic mCMV-specific T cells. We explored mCMV control in the adipose tissue, whose cellular constituents are potentially susceptible to infection. We found that mCMV infects the adipose tissue during the acute phase, causing local inflammation and a lifelong mCMV-specific CD8 T cell immune response. The response consisted largely from non-recirculating, tissue-resident T cells. The infected adipose tissue showed signs of metabolic changes, that may potentially predispose the infected host to metabolic dysregulation as evidenced by hyperglycemia. Accumulation and persistence of mCMV specific non-circulating resident CD8 T cells (Trm) in adipose tissue reveal a likely generalized mechanism of mCMV tissue reservoir control by Trm cells and identify the adipose tissue as a persistent mCMV reservoir, with potential implications for metabolic health.

## Introduction

Cytomegalovirus (CMV) is a ubiquitous beta-herpesvirus that infects most of the worldwide population, with largest prevalence being observed in older adults [1–4]. Acute CMV infection is characterized by system-wide viremia after which latency and lifelong persistence is established in select cells such as CD34+ monocytes and hematopoietic progenitor cells in humans [5–7]. Although CMV infections are generally asymptomatic, untreated infections in utero or amongst the immunocompromised individuals can result in substantial developmental defects, pathology, and death [5,8–10]. However, in immunocompetent patients the substantial and varied (NK, CD8, CD4, and B cells) resources are mobilized to successfully control viral spread and reactivation. One well described arm of anti-CMV immunity, the CD8 T cell compartment, is heavily involved in viral control with up to 5–10% of total CD8s in the blood and secondary lymphoid tissues reactive to CMV antigens during a primary immune response [11–14]. Moreover, in the course of lifelong infection, cycles of latency and reactivation drive an expansion of CD8 T cells, termed memory inflation (MI), that in some cases reaches up to 30–50% of the total memory compartment in mice and men [15,16]. The magnitude of this memory response is largely unparalleled in any other infection and for this reason CMV has been used as a model to understand the effects of MI during immune aging [3,17–20].

Studies of CMV-driven MI, viral dissemination, and persistence throughout the host have largely focused on the spleen, liver, blood, lung, and salivary glands [12,21–24]. It has become clear that the blood contains a major pool of CMV-reactive T effector memory (Tem) cells that presumably scan the vasculature as a bulwark against systemic CMV reactivation and that accumulate with age [3,25]. Tissue control of CMV has been narrowly studied in the context of the lungs and the salivary gland as these sites were shown to harbor mCMV-specific resident non-circulating T cell populations in response to latent virus. However, it remains unclear at this point whether tissue resident memory T cells are universally used to control a persistent pathogen such as CMV *in situ* and to what extent other tissue locations contribute to such a host defense and potential viral latency.

We therefore considered potential locations for where CMV could establish itself and hypothesized that adipose tissue, given several tissue specific properties, could offer a plausible site for infection. Adipose tissue is found at a variety of anatomical locations and consists of multiple cell types including adipocyte progenitors, leukocytes, and stromal cells, some of which have shown susceptibility to mCMV infection [26,27]. Furthermore, the adipose tissue of mouse and man is home to a large proportion of both innate and adaptive immune cells, suggesting that adipose tissue contributes to the mounting of an effective immune response [28–32]. The immune system represented within visceral adipose tissue has been clearly linked to development of diseases of the metabolic syndrome in the context of obesity [28,30,31,33–37]. Given the reported linkage between lifelong CMV infection and metabolic dysfunction in humans we reasoned that mCMV could potentially drive inflammation within adipose tissue that contributes to these phenotypes [20,38]. In further support of this hypothesis, adipocytes have been demonstrated to be susceptible to adenovirus and parasitic infection, raising the possibility that infection could drive insulin resistance and glucose intolerance [39–42]. Furthermore, adipose tissue of HIV/SIV infected humans/monkeys harbored latent virus even after patients were declared virus free following retroviral treatment, suggesting that adipose tissue can provide a safe haven to viruses [43].

Lifelong herpesvirus infections have been studied for their contribution to global host inflammation in the context of frailty and immune aging, but the consequences of these infections on, and the control of infection within adipose tissue have not been investigated. Given the susceptibility of individual cells within adipose tissue to CMV infection we hypothesized that mCMV could infect a cellular constituent of adipose tissue and therefore trigger in an inflammatory immune response *in situ*. We show here that mCMV infects adipose tissue during early peak viremia, followed by infiltration by mCMV-specific CD8 T cells during the acute phase post infection (p.i.). The adipose T cells were specific for mCMV epitopes, with a large fraction of them possessing a Tem phenotype. The presence of inflammatory monocytes was not necessary for the mCMV-specific immune response in adipose tissue, suggesting direct viral spread to adipose tissue during initial infection. mCMV infection and the resulting anti-mCMV CD8 T cells were associated with persistent inflammation within the adipose tissue from very early points during the immune response (days three to five) through the lifespan of the infected host (greater than 450 days). Moreover, infected fat tissue exhibited a decreased production of adiponectin. Finally, an analysis of lifelong-infected animals revealed that the mCMV specific CD8 T cells were *bona fide* Trm (CD69+) cells that exhibit limited exchange with the vasculature based on intravenous staining. The presence of both mCMV and mCMV-specific non-circulating resident CD8 T cells in the adipose tissue for life suggests that Trm cells may be the primary mechanism by which the host controls mCMV tissue reservoirs and their association with inflammation suggest that this interaction may alter metabolic health in infected animals.

## Results

### mCMV infects adipose tissue triggering in an early inflammatory immune response

**Fig 1:**
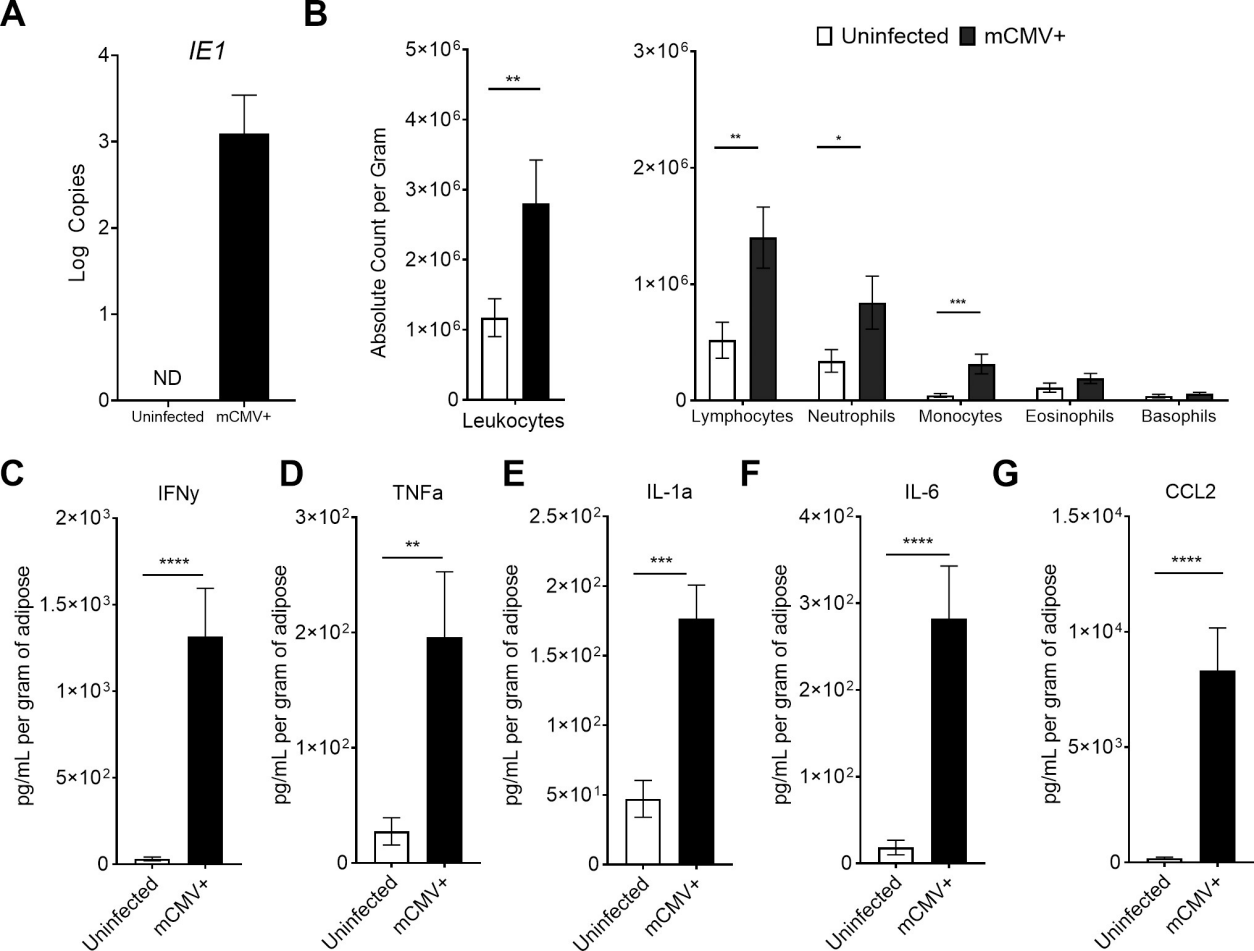
mCMV infects adipose tissue and triggers an immune response. 12-week-old C57BL/6J mice were i.p. injected with 10^5^ pfu of mCMV. (A) Adipose tissue was collected three days post infection into QIAzol reagent and RNA extracted to detect viral gene products *IE1* in 50 ng of total RNA by RT-qPCR and normalized to plasmid. (B) Three days p.i., adipose tissue was processed by collagenase D digestion and stromal vascular fraction was analyzed by hemocytometer. (C-G) Mice infected from three to five days post infection were sacrificed and total adipose homogenized to analyze by BioLegend LegendPlex 13-plex Inflammation Panel. Data are pooled results of two independent experiments. n = 10 uninfected and 10 infected animals total. Error bars represent mean ± SEM. *p < 0.05; **p < 0.01; ***p < 0.001; **** p ≤ 0.0001 by unpaired two-tailed Mann-Whitney U test.

mCMV was reported to infect cells in the adipose tissue [44–46], but the local consequences of this infection *in vivo* have not been well characterized. We first determined if adipose tissue was susceptible to mCMV infection by comparing both RNA (S1A Fig) and DNA (S1B Fig) extracted from total adipose 3 days (d) post infection (p.i.) to uninfected counterparts, we also analyzed the presence of viral genomes in several other tissues and visceral adipose tissue had a two log higher burden at day 3, when compared to subcutaneous adipose, liver, and spleen, likely due to route of infection (S1B Fig). Analysis of RNA transcripts revealed the presence of immediate early (IE) viral gene products, *IE1*, in infected but not uninfected animals (Fig 1A). To determine if the presence of mCMV transcript resulted in an immune response, we measured leukocyte infiltration and found at 3d p.i. a significant increase in the global leukocyte population, which was driven by lymphocytes, neutrophils, monocytes, and eosinophils (as normalized per gram of adipose tissue, Fig 1B). We further characterized the cells participating in this early, 3d p.i., adipose response by flow cytometry and found a significant expansion of NK cells (NK1.1+CD3-; S2A Fig) and an expansion, although not significant, in the macrophage population (F4/80+CD11b+; S2B Fig). Polarization of macrophages to an inflammatory, classically activated M1 phenotype (CD11c+) also trended higher (S2C Fig). We next examined the cytokine content of total adipose tissue homogenate using a flow cytometry-based LegendPlex platform and found significant increases in IFNγ (p = 0.004), TNFα (p = 0.001), IL-1α (p = 0.0007), IL-6 (p<0.0001), and CCL2 (p<0.0001) (Fig 1C–1G) with infection in the adipose tissue on days 3 and 5 p.i. (days 3 and 5 are pooled in the figure). However, at this time point we did not observe any significant changes in the amounts of GM-CSF, IFNβ, IL-1β, IL-12, IL-17A, IL-23, or IL-27. Therefore, during the early acute timepoints of infection, we found evidence of virus transcription, cellular and cytokine immune responses in the total adipose tissue.

### mCMV-specific CD8 T cells infiltrate adipose tissue during acute infection

**Fig 2:**
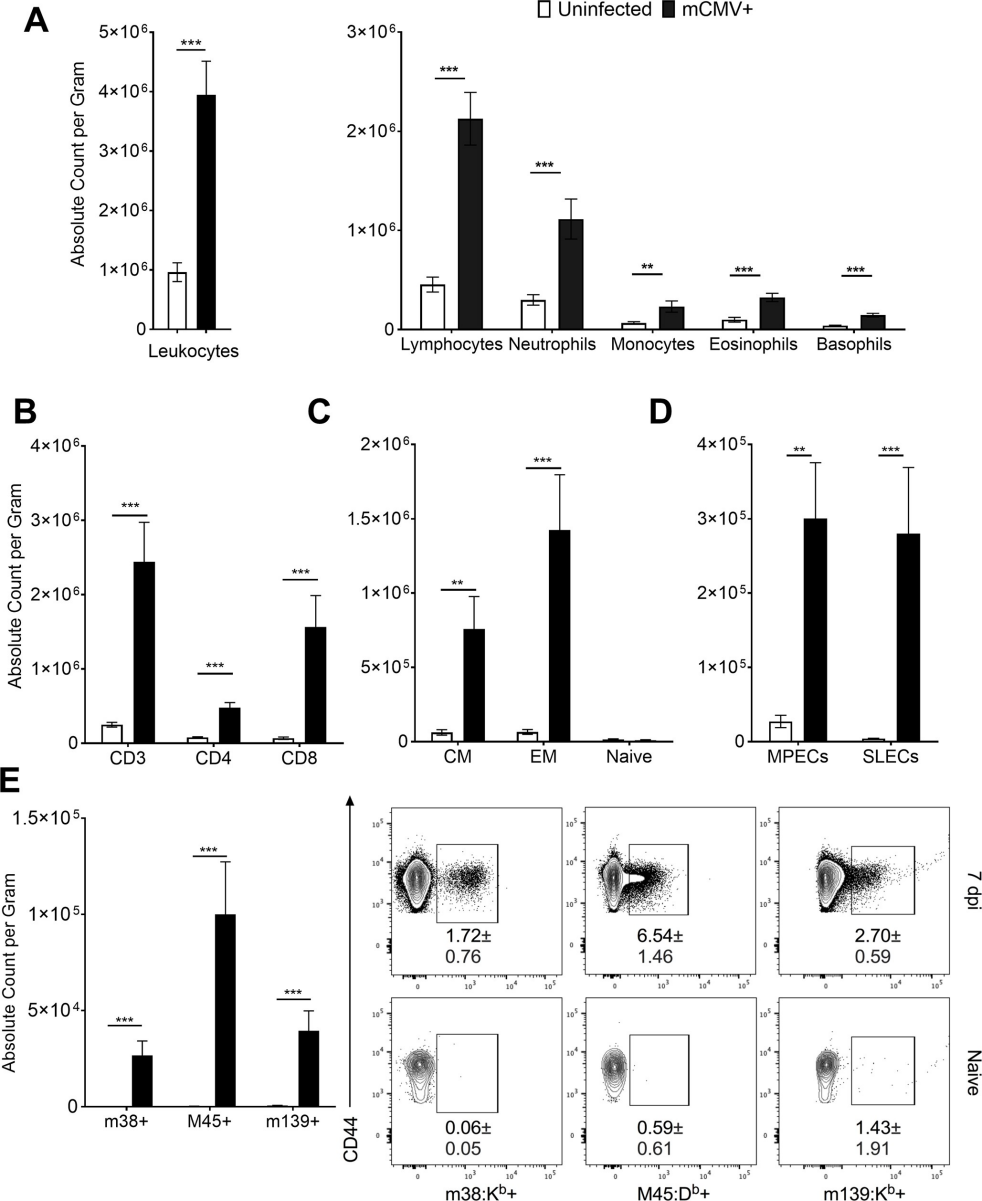
Adipose tissue is infiltrated by mCMV-specific T cells. 12-week-old C57BL/6J mice were i.p. injected with 10^5^ pfu of mCMV and sacrificed on d7 p.i.. Stromal vascular fraction was analyzed by Drew Scientific HemaVet 950 and flow cytometry. (A) Total leukocytes were quantified by hemocytometer. (B) Flow cytometry analysis was used to quantify absolute numbers per gram of adipose tissue of CD3 T cells and subsetted into CD4 or CD8 pools. (C) CD8 T cells were phenotyped based on expression of CD62L and CD44 and quantified. (D) CD44+ CD8 T cells were analyzed for expression of KLRG1 and CD127 to quantify number of MPECs and SLECs. (E) CD44+ CD8 T cells were analyzed for mCMV specificity by tetramer staining. Data are pooled results of two independent experiments. n = 9 total infected and 6 uninfected animals total. Frequencies shown in the dot plots represent SD. Error bars represent mean ± SEM. *p < 0.05; **p < 0.01; ***p < 0.001; **** p ≤ 0.0001 by unpaired two-tailed Mann-Whitney U test.

Following infection, mCMV replicates systemically, leading to detectable host viremia that is resolved within days of infection. It is possible that the inflammation observed in the adipose at this early timepoint could resolve during reduction of the viral load. On 7d p.i. we were able to detect mCMV viral products (S1A Fig). Given this detection we wondered if there was a continued immune response within adipose tissue. We therefore quantified inflammation during the peak adaptive immune response at the same time, again by characterizing the cellular content of the stromal vascular fraction (SVF). The global leukocyte population of infected animals (Fig 2A, left) remained elevated, driven by lymphocytes, neutrophils, monocytes, eosinophils, and basophils (Fig 2A, right). Contributing to this expansion were NK cells which were still significantly elevated (S2A Fig). Total macrophage population became significantly expanded in infected adipose tissue at this time as well (S2B Fig). Approximately half of all leukocytes detected in adipose tissue at this time were lymphocytes, and a majority of these were CD3 T cells, with significant increases in both the CD4 and CD8 (p = 0.0004) populations (Fig 2B). Both the central memory (CD44+CD62L+; p = 0.012) and effector memory (CD44+CD62L-; p = 0.004) populations were significantly increased in infected animals, whereas, as expected, there was no change in the total numbers of naïve (CD44- CD62L+) T cells (Fig 2C). Amongst the memory subsets we found roughly equivalent numbers of memory-precursor effector cells (CD127+KLRG1-; MPECs; p = 0.0028) and short-lived effector cells (CD127-KLRG1+; SLECs; p = 0.0004), with both populations significantly increased in infected animals. We next asked whether these T cells were specific for mCMV antigen or were accumulating due to perhaps inflammatory stimulation in a non-specific manner. Peptide:MHC (pMHC) tetramer staining revealed a significant expansion of T cells specific for the acute, non-inflationary immunodominant epitope M45 (p = 0.0004), with smaller, but also highly significant expansion of CD8 T cells specific for inflationary epitopes m38 (p = 0.0004) and m139 (p = 0.0004) (Fig 2E). Given an influx of antigen specific T cells we also tested whether the cytokine milieu was still altered, and found, by LegendPlex, significant increases in the protein levels of IFNγ (S3A Fig; p = 0.004) and CCL2 (S3B Fig; p = 0.0005), but not of GM-CSF, IFNβ, IL-1α, IL-1β, IL-6, IL-10, IL-12, IL-17A, IL-23, IL-27, or TNFα. In addition to these protein analyses we found several inflammatory transcripts that were significantly upregulated at this time point, including *Cd3e*, *Ifng*, *Cxcr3*, *Ccr5*, *Casp1*, and *Adgre1*, indicative of a myeloid and T lymphocyte infiltration (S4 Fig).

Adipose tissue inflammation has been demonstrated to alter adipocyte derived cytokines, adipokines, during the onset and establishment of obesity [47,48]. We therefore examined the production of two well described adipokines, leptin and adiponectin. We found that adiponectin, which is decreased during inflammation-induced by obesity [49], was significantly decreased in infected animals (S5A Fig; p = 0.0186), whereas, we found no change in the amount of leptin, which is positively correlated with body weight (S5B Fig) [50]. No significant change in total leptin, however, comes as no surprise as we saw no significant change in total epididymal fat pad or body weight during the lifespan of infection (S6 Fig). These data suggest that inflammation driven by infection and influx of immune cells can trigger a secretory response by adipocytes. Taken all together, these data indicate that mCMV infection results in a CD8 T cell response detectable in adipose tissue at 7d p.i.

### Adipose tissue accumulates CD8+ mCMV-specific CD8 T cells regardless of the route of infection or the presence of CCR2+ cells

**Fig 3:**
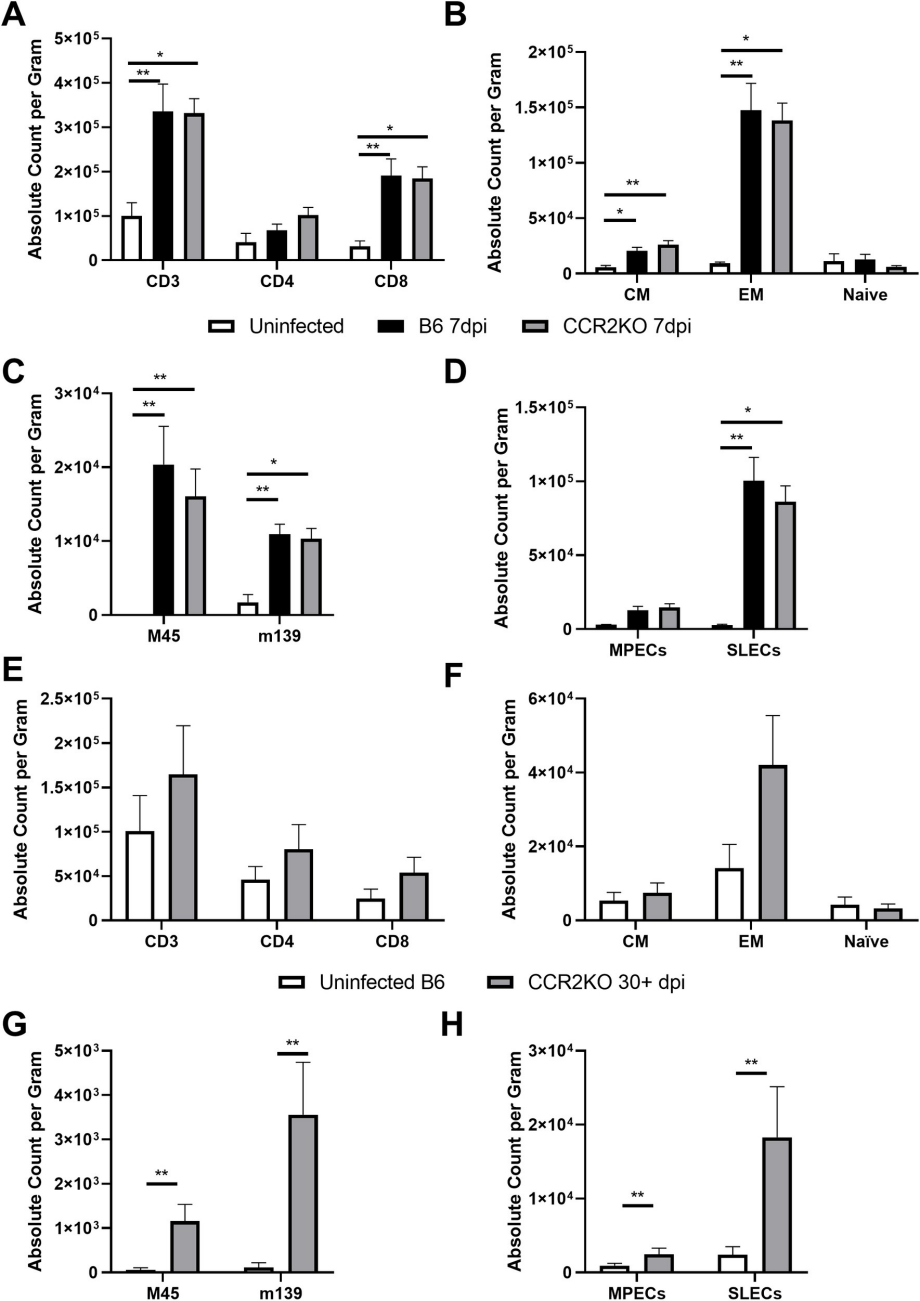
Adipose tissue accumulates CD8+ mCMV-specific CD8 T cells regardless of the route of infection or the presence of CCR2+ cells. 12-week-old C57BL/6J and CCR2^-/-^ mice were infected with 10^5^ pfu of mCMV via the footpad and sacrificed at 7d p.i.. Stromal vascular fraction was analyzed by flow cytometry. (A–D) 7d p.i. (A) Total number of T cells and subsetted into CD4 and D8 pools. (B) CD8 T cells were phenotyped based on expression of CD62L and CD44 and quantified. (C) CD44+ CD8 T cells were analyzed for mCMV specificity by tetramer staining. (D) CD44+ CD8 T cells were analyzed for expression of KLRG1 and CD127 to quantify number of MPECs and SLECs. (E–H) 30d + p.i. (E) Total number of T cells and subsetted into CD4 and D8 pools. (F) CD8 T cells were phenotyped based on expression of CD62L and CD44 and quantified. (G) CD44+ CD8 T cells were analyzed for mCMV specificity by tetramer staining. (H) CD44+ CD8 T cells were analyzed for expression of KLRG1 and CD127 to quantify number of MPECs and SLECs. Data are pooled results of two independent experiments. (A–B) n = 5 uninfected, 10 C57BL/6J infected, 10 CCR2^-/-^ infected animals total. (E–H) n = 6 uninfected and n = 6 infected. Error bars represent mean ± SEM. *p < 0.05; **p < 0.01; ***p < 0.001; **** p ≤ 0.0001. 7d p.i. are tested by Kruskal-Wallis with Dunn’s multiple comparisons and 30d+ p.i. by unpaired two-tailed Mann-Whitney U test.

CCR2+ cells are believed to be the major carriers of mCMV, involved in virus dissemination [51,52]. Moreover, intraperitoneal injection, used in our experiments, may result in an indiscriminate and non-physiological distribution of the virus, including to the epididymal adipose tissue. To assess whether the virus exhibited true tropism for adipose tissue or perhaps infected the immunological constituents of adipose tissue regardless of the route of infection, we infected animals using 10^5^ pfu of mCMV via the footpad (f.p.) route of infection. We considered two possibilities in which this receptor could be required 1) that CCR2 is required for homeostatic seeding of CCR2+ infected cells from the periphery into the adipose tissue, resulting in “reinfection” of adipose; or 2) that CCR2+ patrolling cells are not required to maintain antigen and therefore the presence of T cells *in situ*. We also wondered whether CCR2+ cells were needed to spread the virus during normal homeostatic seeding of adipose. To that effect, we infected both C57BL/6 and CCR2^-/-^ mice [53] via the f.p. route. Surprisingly, when we analyzed adipose tissue at days 3 and 7 p.i. of C57BL/6 and CCR2^-/-^ mice we were unable to detect viral product in either strain of mouse, perhaps indicative of virus being below the limit of detection or unable to spread to adipose tissue through this route. However, at 7d p.i. when we analyzed adipose tissue by flow cytometry, we found a significant increase in CD3 T cell numbers driven by a significant expansion of CD8 T cell in adipose of both wildtype and CCR2^-/-^ mice (Fig 3A). Of interest, we saw a very limited expansion of central memory CD8 T cells (CD62L+CD44+) in the adipose when infected through this route in both wildtype and CCR2^-/-^ mice, while the significant expansion of effector memory cells (CD62L-CD44+) was not diminished (Fig 3B). When we assayed tetramer specificity, we found a significant expansion in both acute, M45, and inflationary, m139, epitopes (Fig 3C). Finally, we analyzed the MPEC and SLEC populations and found that MPECs did not significantly expand whereas SLECs did (Fig 3D). These results suggest that mCMV infection, perhaps even at extremely low levels of viral burden, leads to the accumulation of mCMV-specific T cells in the adipose tissue regardless of the route of infection or in the presence of CCR2 (and, presumably, of CCR2+ cells). However, it should be noted that memory precursors and central memory cells appear to be insensitive to this route of infection (Fig 3B and Fig 3D).

We then wished to determine if there was a requirement for CCR2 to maintain viral-specific T cells within adipose tissue given the lack of central memory and memory precursors through f.p. infection. We therefore infected as before, via the footpad route, CCR2^-/-^ mice and quantified the mCMV-specific T cell in adipose at an early memory timepoint post infection, greater than 30d p.i.. We found an overall diminished T cell population within the adipose of infected animals with no single T cell subset being significantly increased during infection (Fig 3E). Just as in the 7d p.i. timepoint, we saw no significant expansion of central memory CD8 T cells and found a trend of increase in the effector memory pool (Fig 3F). However, even in the absence of this expansion of global T cell populations, there was a significant increase in the mCMV M45- and m139-specific subsets (Fig 3G) as well as an expansion of both MPECs and SLECs at this time (Fig 3H).

Taken together, we conclude that the f.p. route of infection leads to undetectable viral load in adipose tissue, which results in no expansion of central memory and memory precursor CD8 T cells. Nonetheless, effector and short-lived effector populations do expand, albeit to a lesser degree than i.p.. Finally, tetramer specific T cells still arise and persist in adipose tissue regardless of the absence of CCR2 and route of infection.

### Long-term persistence of tissue-resident mCMV-specific T cells and of inflammation in the infected adipose tissue

**Fig 4:**
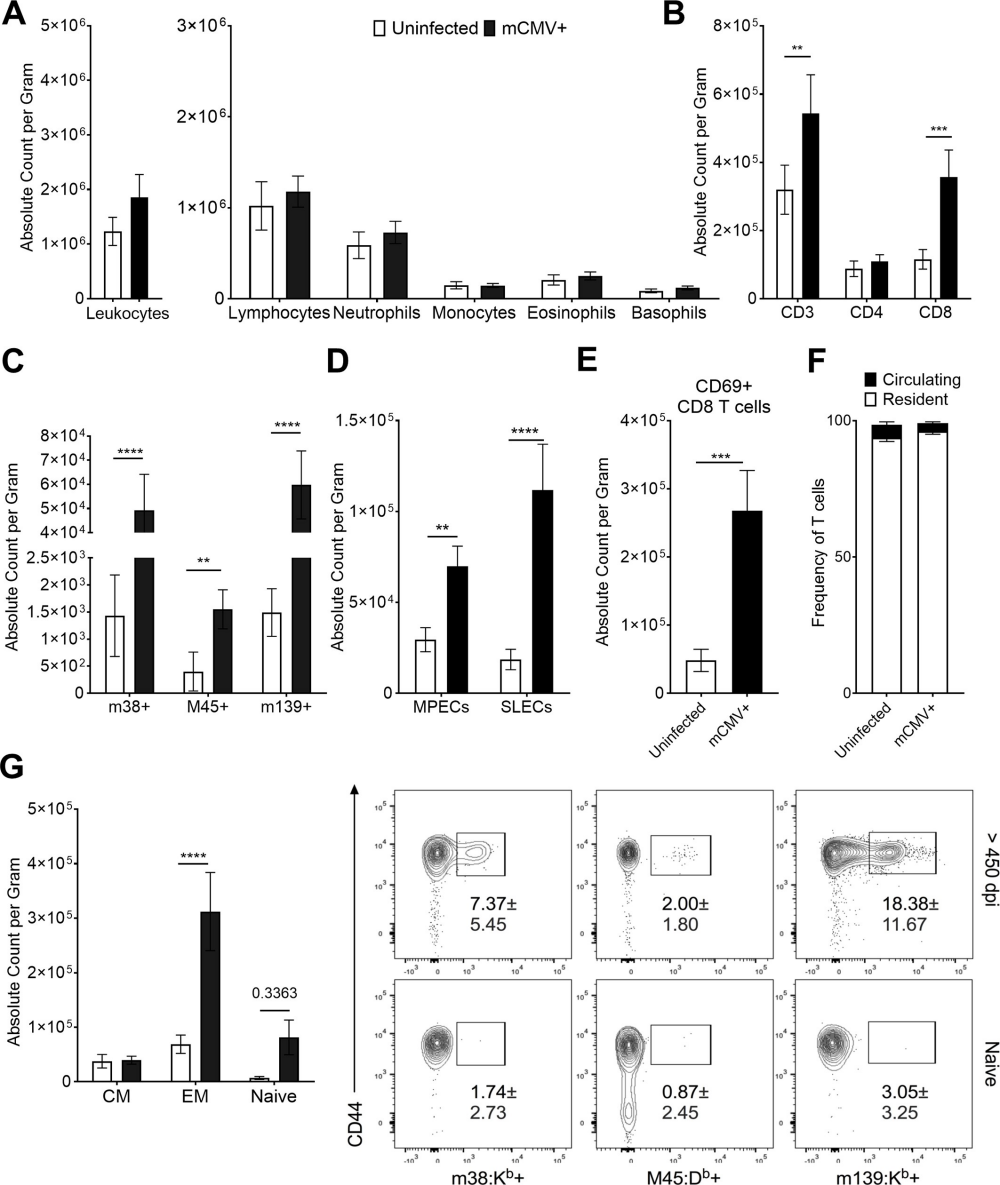
mCMV-specific T cells are maintained in adipose tissue for the lifespan of infection. 12-week-old C57BL/6J mice were i.p. injected with 10^5^ pfu of mCMV and sacrificed >450d p.i. Stromal vascular fraction was analyzed by Drew Scientific HemaVet 950 and flow cytometry. (A) Total leukocytes were quantified by hemocytometer. (B) Flow cytometry analysis was used to quantify absolute numbers per gram of adipose tissue of CD3 T cells and gated on CD4 or CD8. (C) CD8 T cells were phenotyped based on expression of CD62L and CD44 and quantified. (D) CD44+ CD8 T cells were analyzed for expression of KLRG1 and CD127 to quantify number of MPECs and SLECs. (E) Total CD8 T cells were analyzed for surface expression of CD69. (F) Lifelong infected animals were injected i.v. with 3 μg of CD45 antibody to determine tissue residency of T cells. Frequency of *in* vivo and *ex* vivo stained animals is shown. (G) CD44+ CD8 T cells were analyzed for mCMV specificity by tetramer staining. (A-E and G) Data are pooled results of two independent experiments. n = 20 uninfected animals and 19 infected animals total. (F) Data are pooled results of two independent experiments with an n = 9 uninfected animals and n = 9 infected animals total. Frequencies shown in the dot plots represent SD. Error bars represent mean ± SEM. *p < 0.05; **p < 0.01; ***p < 0.001; **** p ≤ 0.0001 by unpaired two-tailed Mann-Whitney U test.

To assess the impact of mCMV infection in the adipose tissue over the lifespan, we analyzed the fat pads of lifelong infected animals. During the lifelong time points of infection (>450d p.i.) we were unable to detect mCMV RNA. We therefore looked at the maintenance of mCMV genomes in the adipose tissue of infected animals via qPCR. To get a better resolution on viral genome loads, we initially compared CD45- non-hematopoietic and CD11b+CD45+ myeloid cell pools after FACs sorting (S7A Fig). Since the CD45- fraction showed a trend towards a higher mCMV genome burden (S7B Fig), we focused on these cells in subsequent kinetic analysis. mCMV genomes persisted in the adipose CD45- tissue at comparable levels from 90 to approximately 300+ days post infection, suggesting a lifelong presence of the latent and/or reactivating virus in the adipose tissue of infected animals (S7C Fig). We next went on to characterize the immunological response at these late time points. We found that total leukocyte counts in the adipose tissue were no longer significantly elevated, showing just a trend (Fig 4A). However, there remained a significant increase in CD3 T cells in infected animals (p = 0.0407), which was entirely driven by a robust expansion of CD8 T cells (Fig 4B; p = 0.0011), with a dominant and significant increase in Tem CD8 cells (Fig 4C; p<0.0001), and a stronger skewing towards SLECs (p<0.0001) over MPECs (p = 0.0069) compared to the acute (7d p.i) infection (Fig 4D). At this point NK cells and macrophage populations in infected animals mirrored that of their aged-matched counterparts (S2 Fig), suggesting that perhaps viral control of mCMV in adipose at these later time points is more reliant upon T cells.

To examine whether CD8 T cells in the adipose tissue were recirculating from the systemic pool, we performed two experiments. First, we analyzed expression of CD69 on CD8 T cells. This molecule, often used as a marker of immediate activation, is an antagonist of the S1P1 receptor, leading to the retention of T cells in their specific tissue [54]. We found that 75% of all CD8 T cells expressed CD69 in infected animals, a significant increase compared to that of their uninfected counterparts (Fig 4E; p = 0.0015). We also analyzed the dual expression of CD103e, which has been used to define tissue resident cells in other tissues [54,55] and found no significant differences between its expression on CD8 T cells in infected and uninfected animals (S8 Fig). Second, to independently test whether and how many CD8 T cells in the adipose tissue may be of resident memory type, we assessed their accessibility to a systemic anti-CD45 antibody injected into the vasculature *in vivo*, as a measure of their vascular vs. tissue-resident location. We injected an Alexa Fluor 700 labeled anti-CD45 antibody intravenously (i.v.) into lifelong infected animals, harvested the adipose tissue 5 min later, as previously described, to determine the extent of T cell tissue residency [56]. We found that approximately 95% of all T cells in adipose tissue of infected (as well as uninfected) animals stained only with the *ex vivo* antibody and therefore could be classified as resident to adipose tissue (Fig 4F).

To ascertain that T cells in lifelong infected animals are specific for mCMV antigens, we repeated the tetramer staining as performed in earlier timepoints. We found that a majority of CD8 T cells within adipose tissue remained specific for mCMV tetramers, with an expected and significant expansion of T cells specific for the inflationary T cells epitopes, m38 (p<0.0001) and m139 (p<0.0001). A much smaller, but also significantly expanded population was specific for the acute M45 epitope (p = 0.0018), possibly indicative of recent viral reactivation (Fig 4G). Taken together, these data demonstrate that mCMV-specific CD8 T cells are maintained within the adipose tissue for the lifespan of infection, as *bona fide* Trm cells.

**Fig 5:**
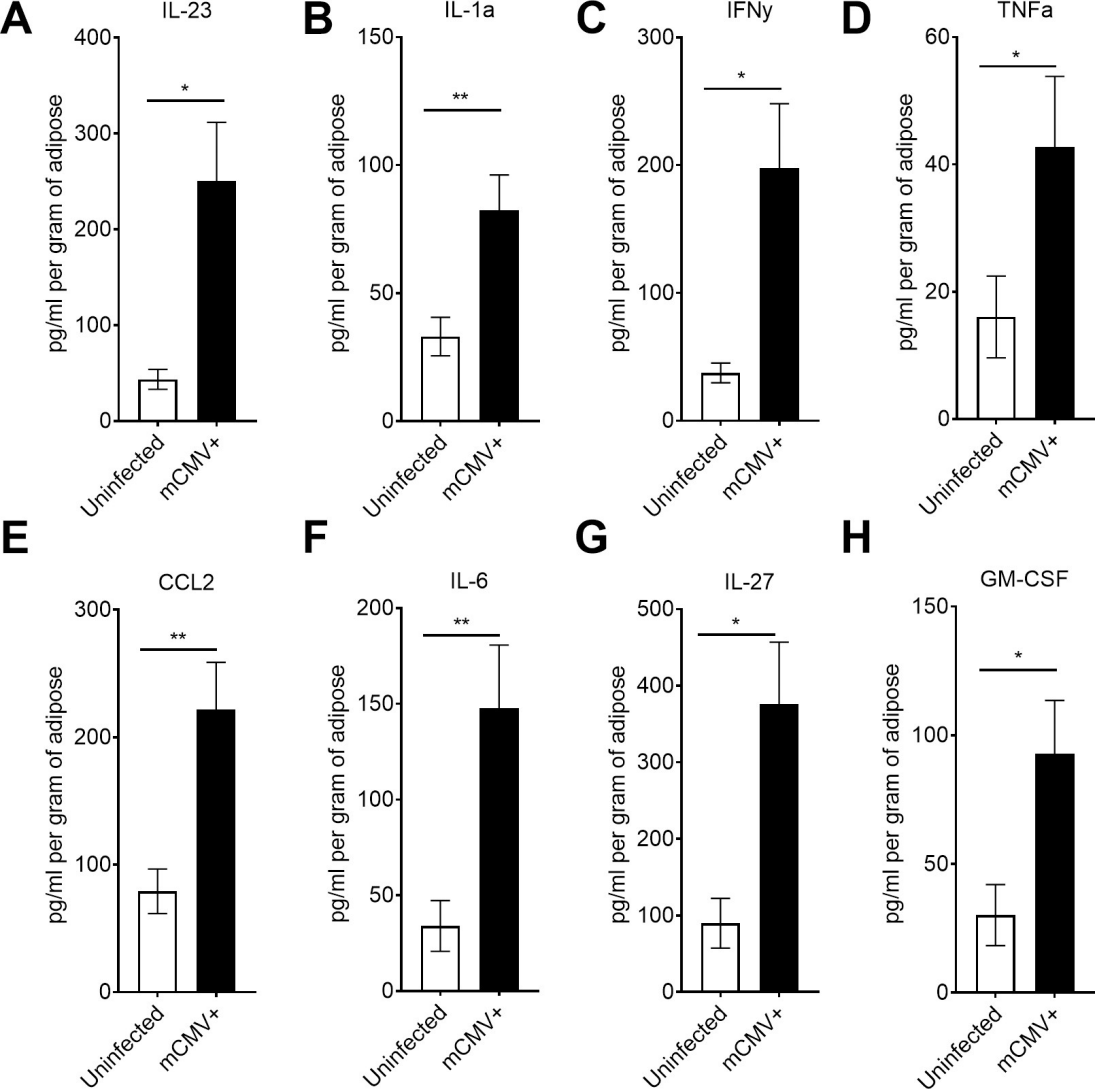
Lifelong mCMV infection results in inflammation in the adipose tissue. 12-week-old C57BL/6J mice were injected with 10^5^ pfu of mCMV and sacrificed at greater than 450d p.i.. Total adipose tissue was homogenized and analyzed by BioLegend LegendPlex. (A) IL-23 (B) IL-1α (C) IFNγ (D) TNFα (E) CCL2 (F) IL-6 (G) IL-27 and (H) GM-CSF were all statistically increased. Data are pooled results of two independent experiments. n = 9 uninfected and 10 infected animals total. Error bars represent mean ± SEM. *p < 0.05; **p < 0.01; ***p < 0.001; **** p ≤ 0.0001 by unpaired two-tailed Mann-Whitney U test.

The presence of phenotypically active mCMV specific T cells in adipose tissue provides evidence of a continued surveillance against mCMV. Next, we investigated whether this significant presence of mCMV-specific Trm cells within adipose tissue over the lifespan may be associated with persistent inflammation. We found that IL-23 (p = 0.0201), IL-1α (p = 0.0071), IFNγ (p = 0.0113), TNFα (p = 0.0258), CCL2 (p = 0.0083), IL-6 (p = 0.0083), IL-27 (p = 0.0109), and GM-CSF (p = 0.0175) were all significantly elevated in lifelong infected adipose tissue when compared to uninfected age matched controls (Fig 5A–5H). By contrast, IFNβ, IL-1β, IL-10, IL-17A, and IL-12 did not exhibit significant changes when compared to uninfected animals. These data indicated that adipose tissue is a site of lifelong accumulation, or maintenance, of mCMV-specific Trm cells that exhibit phenotypic evidence of recent antigenic stimulation, and that this correlates with an inflammatory cytokine response over the entire lifespan of the host.

### Persistent mCMV infection is correlated with hyperglycemia

**Fig 6:**
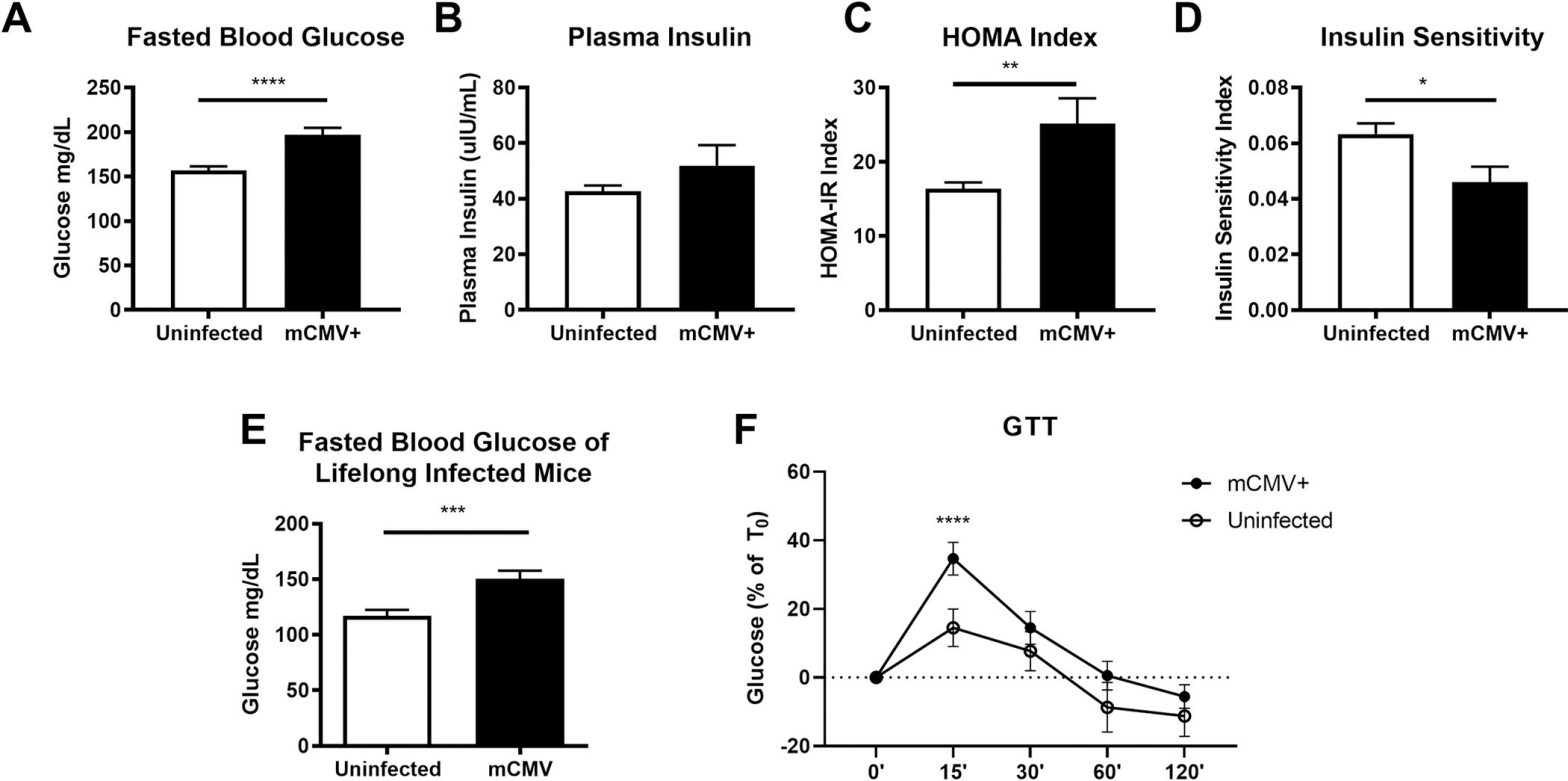
Chronic mCMV infection is correlated with hyperglycemia. 12-week-old C57BL/6J mice were infected with 10^5^ pfu of mCMV by the i.p. route. Prior to blood glucose measurements via tail nick or collection of blood for plasma via retro-orbital bleed mice were fasted for at least 7 hours. (A) Fasted blood glucose of mice infected between 10 and 12 weeks as measured by Bayer Contour Next EZ Glucose Meter. (B) Plasma insulin concentration measurements by ELISA of mice infected between 6 and 10 weeks. (C) HOMA-IR and inverse measurement (D) Insulin sensitivity index. (E) Fasted blood glucose of lifelong (>450d p.i.) infected mice. (F) Percent change in fasted blood glucose of lifelong infected mice challenged with i.p. glucose bolus. Data are pooled results of two to four independent experiments. For the 10 to 12-week experiments there were an n = 14 uninfected and n = 10 infected animals total. For (D) experiments there were n = 23 uninfected and 35 infected animals total. (F) are pooled results of two independent experiments with n = 18 infected and n = 10 uninfected mice total. Error bars represent mean ± SEM. *p < 0.05; **p < 0.01; ***p < 0.001; **** p ≤ 0.0001 by unpaired two-tailed Mann-Whitney U test.

Adipose tissue inflammation associated with obesity has been clearly linked with multiple phenotypes of the metabolic syndrome, including glucose intolerance and insulin resistance [44,57,58]. Based on the observed increase in inflammatory cytokines and cytotoxic T cells in lifelong infected animals we hypothesized that infected mice could exhibit an altered metabolic profile. Indeed, we found that between ten- and twelve-weeks post infection there was an elevation of fasted blood glucose in infected animals (Fig 6A) with no significant differences between infected and uninfected animals in plasma insulin levels (Fig 6B). To determine if the hyperglycemia was correlated with increased adiposity of infected animals, we longitudinally followed mice and analyzed the weight of their fat pads and found no significant change in fat pad weights between infected and uninfected mice (S6A Fig). When we calculated the homeostasis model assessment insulin resistance (HOMA-IR) index and found that infected animals exhibited significant elevation of this index compared to uninfected animals (Fig 6C; p = 0.0155). Conversely, the inverse of HOMA-IR, the insulin sensitivity index, expectedly suggested that infected animals were less sensitive to insulin than uninfected controls (Fig 6D; p = 0.0155). At >450 days post infection, significantly elevated levels of blood glucose were still observed in infected animals (Fig 6E; p = 0.0006) and this occurred in the absence of a significant increase in body weight (S6B Fig). This elevation in fasted blood glucose appeared to be dependent on mature CD8 T cells as we found no significant differences between the fasted blood glucose of chronically infected and uninfected mice lacking beta-2-microglobulin (B2m KO) (S9 Fig).

Based on the results of the HOMA-IR analysis of mice between ten- and twelve-weeks post infection we analyzed more broadly the metabolic system of lifelong infected animals. First, we determined the extent to which infected and uninfected animals clear a bolus of glucose by i.p. challenge. We found that mCMV infected animals did not clear glucose from the blood as quickly as uninfected counterparts (Fig 6F). We next tested whether the HOMA-IR was an accurate representation of insulin resistance in our model. Therefore, we i.p. challenged with fast acting insulin to determine insulin sensitivity and found no significant differences between infected and uninfected animals (S10A Fig). Finally, we wondered if elevated fasted blood glucose indicated a hyperactive gluconeogenesis driven by the liver. We therefore challenged mice with sodium pyruvate i.p. to determine liver sensitivity to infection and found no difference in gluconeogenesis in infected and uninfected animals (S10B Fig).

Taken together these data are consistent with recently published work that suggests alterations in glucose tolerance and insulin sensitivity in mice acutely infected with mCMV and influenza infection of mice being fed a high fat diet [59]. When we measured adiponectin expression in adipose tissue homogenate, we expected decreased amounts of total protein as we saw in the acute time point post infection, however we found no significant change in the amount of adiponectin protein in uninfected and lifelong infected animals (S11 Fig), perhaps indicating an age related decrease in adiponectin expression that may mask changes induced by infection. Overall, we found a clear initial alteration in the glycemic profile of mCMV-infected mice following infection that appears to be driven by delayed glucose clearance in infected animals and is possibly dependent upon mature T cells. Additional studies will be required to mechanistically extend these data, one is tempted to speculate that mCMV may make animals susceptible to clinical metabolic changes pending action of other environmental stressors, including diet, as previously published, and aging in our model.

## Discussion

CD8 T cell immunity against mCMV infection has been extensively studied in the context of inflationary memory T cell expansion in the blood, as well as in the lungs (as the port of CMV entry) and salivary gland (as the site of intense primary CMV replication). Results of these studies have suggested that the blood contains a large pool of CMV-specific circulating Tem cells, guarding against potential systemic reactivation, whereas both the site of primary entry (lungs) and extensive initial replication and shedding (salivary gland) contain Trm cells standing guard against potential reinfection (lung) and/or local reactivation (lung and salivary gland). CMV is believed to infect many cells, but to establish latency only in very few [7,60,61]. In that context and in the context of an early and lifelong CMV infection and immunity, we know very little about CMV-specific CD8 T cell immunity and control in other tissues. For example, one fundamental question remains on whether the large systemic circulating CD8 T cell pool is responsible for the control of other potential sites of latency and reactivation.

Recent studies show that white adipose tissue is enriched in leukocytes, including a significant population of memory T cell populations even in mice housed under specific-pathogen free conditions [31,62,63]. Furthermore, it has been demonstrated that pathogen-specific T cells can arise in both mesenteric and epididymal adipose tissue following bacterial and parasitic infection [43,62]. It has also been demonstrated that murine adipose tissue can harbor infectious mCMV as demonstrated by plaque assay and microscopy during early time points post infection [45,59,64]. We show here that adipose tissue is an early site of infection which leads to generalized inflammation, maintains viral genomes for the lifetime, and possesses a sustained antigen-specific adaptive immune response. We found that mCMV-specific Tem CD8 T cells dominated the immune response early, and this response was maintained for life. Moreover, both phenotypic and functional (vascular accessibility) data were consistent with the Trm nature of fat-residing CD8 T cells.

Several groups have demonstrated that mucosal tissues, such as the salivary gland and lungs, are home to non-recirculating T cells that respond to mCMV [65–67]. This is largely believed to be in response to mCMV utilizing mucosa as a means for spread through saliva, breast milk, urine, and vaginal fluids [68]. Authors have suggested two potential methods that result in T cell accumulation in the lung and the salivary gland: 1) T cells primed in the periphery traffic to these locations; and/or 2) viral antigens, (even in the absence of full replication as demonstrated by experiments conducted with replication-incompetent mCMV) are presented *in situ*, evoking cytokine and chemokine cues that maintain memory T cells after original antigenic stimulation. We interpret our data as indicative of continual maintenance of memory T cells *in situ*. However, this raises two questions. First, why would adipose tissue be evolutionarily advantageous for mCMV infection? HCMV alters the lipid metabolism of infected cells [69,70] and given the high density of lipids within adipocytes it is possible that adipocytes or their progenitors and even fibroblasts could provide significant sources of lipids and therefore become prime targets for infection. Furthermore, different cells of the adipose tissue, including adipose tissue-derived stem cells [71], have been shown to be susceptible to CMV infection. Second, if viral antigens are not being presented within adipose tissue, why would the immune system divert a lifelong T cell population to this site? Other groups have suggested that the presence of memory T cells in the fat would be expected given the anatomical location of adipose tissue with respect to lymphatic organs, the gut, and the vasculature to provide clean-up for any antigenic leakage from these tissues. We show that fat-residing CMV-specific T cells are phenotypically activated, suggesting recent antigenic stimulation. That would support the hypothesis of antigenic presentation *in situ*, which may be supported by the PCR detection of viral gene products during the 10 months post infection period. While, at present, we cannot formally exclude that CMV antigens may indeed leak from these proximal tissues, we consider such a possibility less likely, given the tight temporal regulation of mCMV antigen expression. An alternative possibility would be that trafficking cells harboring CMV, such as inflammatory monocytes, could potentially be continually seeding the adipose tissue and that this would help maintain the mCMV-specific Trm cells. Against that possibility, we found that CCR2^-/-^ mice, infected via the footpad route, as an attempt to isolate initial replication as much as possible, also exhibited significant accumulation of mCMV-specific T cell population in the fat, suggesting either cell-free spread or a non-monocyte cell-associated virus, below our limit of detection at this time, as drivers of T cell accumulation in the adipose tissue. Based on the preponderance of evidence, we favor the scenario whereby a persistent, *bona fide* latency established by mCMV within adipose tissue drives the accumulation of CD8 Trm cells.

Inflammation within adipose tissue has been widely investigated for its role in the development of metabolic syndromes [58]. In our experiments, mCMV infection resulted in inflammation within adipose tissue in the absence of obesity. The influx into the adipose tissue by leukocytes and specifically CD8 Tem/rm cells could potentially alter the metabolic profile of infected mice. When we measured glucose and insulin changes in infected animals, we did not observe any changes in fasted blood glucose until animals were ten to twelve weeks post infection, by which time we did not detect any difference in systemic insulin levels. This difference was maintained in lifelong infected animals, showing a significant elevation in the fasted blood glucose of infected animals, but with no statistical difference in the total weight, at end of life, or longitudinal differences in fat pad hypertrophy or atrophy. These observations are consistent with recent data demonstrating that the production of IFNγ in response to mCMV and influenza infection in a model of dietary-induced obesity was the “tipping” point in the manifestation of insulin resistance [59]. Furthermore, we find that neither gluconeogenesis nor reduced insulin sensitivity were responsible for elevated fasted blood glucose. Rather, we believe that infection potentially alters systemic glucose control, an issue that will require further experimentation. Thus, our finding could provide one potential mechanism to link epidemiological data in humans showing that HCMV infection increases the risk of developing atherosclerosis, insulin resistance, and other metabolic diseases [72–76]. In that scenario, we speculate that CMV infection alone could increase one’s risk for developing metabolic disorders, but that additional environmental factors are required, such as diet, other infections, and aging; to what extent this interplay is dependent upon adipose tissue remains to be established.

Our data identify adipose tissue as a potential reservoir for mCMV genomic persistence, through our detection of viral products at 10 months post infection. mCMV infection clearly leads to the continuous stimulation of antigen-specific CD8 T cells that take up residency within adipose tissue, based upon phenotypic data. Trm cells are maintained for the lifetime of infection and likely contribute to an inflammatory environment within adipose tissue. These data reveal a strategy by which the adaptive immune system controls mCMV in tissues and provide insights that could mechanistically link mCMV infection of the adipose tissue to metabolic dysfunction, that may depend on additional metabolic and environmental stressors, such as aging and diet.

## Methods

### Ethics statement

Mouse studies were carried out in strict accordance with the recommendations in the Guide for the Care and Use of Laboratory Animals of the National Institutes of Health. Protocols were approved by the Institutional Animal Care and Use Committee at the University of Arizona (IACUC #08–102, PHS Assurance Number: A3248-01). Footpad injections were performed under isoflurane anesthesia. Euthanasia was performed by isoflurane overdose. Animal experiments performed at Helmholtz Centre for Infection Research (Braunschweig, Germany) were approved by Lower Saxony State Office of Consumer Protection and Food Safety under the license number 33.9–42502-04-14/1712.

### Mice and lifelong MCMV infection

Ten-week-old adult C57BL/6J and congenic CD45.1 (B6, H-2b), B2m KO, and CCR2^-/-^ male mice were purchased from The Jackson Laboratory. At 12 weeks of age, adult mice were infected with 10^5^ pfu of mCMV intraperitoneally or via footpad, both routes produce overlapping data, (Smith strain, originally obtained from M. Jarvis and J. Nelson, Oregon Health and Science University, Portland, OR, passage 3 on M210B4 cells. Mice were maintained under specific pathogen-free conditions in the animal facilities at the University of Arizona and at Helmholtz Centre for Infection Research (Braunschweig, Germany).

### Isolation of stromal vascular fraction

Animals were sacrificed by isoflurane overdose. White adipose tissue from the epididymal fat pad was excised, weighed, and cut in small pieces using forceps and scissors. Cut pieces were resuspended in DMEM containing 2 mg/ml Collagenase D (1mL solution per 0.5 grams of adipose tissue) and incubated for 30 minutes at 37°C with shaking. Digestion suspensions were thoroughly vortexed and centrifuged at (800*g* for 5 min). Adipocyte fraction and liquid interphase was sterile vacuumed away from pellet, which was resuspended in DMEM containing 5% BSA and pushed through a 70 μm nylon mesh filter to remove remaining cell debris. Cells were centrifuged and resuspended in 250 ul PBS containing 1% BSA. 50 ul of resuspension was used to calculate cell counts per gram of adipose tissue and the remaining used for flow cytometry. Numerical quantification of single cell suspensions was carried out using Drew Scientific HemaVet 950.

### Real-time PCR quantification of viral RNA load in tissues

Adipose tissue was collected into a microcentrifuge tube filled with 1 mL of Qiazol and autoclaved glass beads and then snap frozen in liquid nitrogen. Samples were thawed and homogenized using a bead beater for two 2-minute cycles. RNA was extracted using Qiagen RNeasy Lipid Tissue Mini Kit per the manufacturer’s protocol. Reverse transcription was carried out using Sensiscript RT Kit per manufacturer instructions. Amplification of cDNA was performed using SYBR Green Master Mix on an ABI 7300. Standard curve was generated using plasmid gifted from Wayne Yokoyama, MD, Washington University in St. Louis. Primer set were gifted by Chris Benedict, PhD, La Jolla Institute of Immunology [72]. For applications utilizing the RT2 Miniarray Profiler samples were treated as above, analyzed for RNA Integrity Number (RIN) by the University of Arizona Genetics Core Facility on an Agilent Bioanalyzer 2100. Following manufacturer protocol only samples with a RIN greater than 7 were used. Analysis was carried out through Qiagen’s Data Analysis Center.

### Real-time PCR quantification of viral genome load in tissues

The liver, spleen, subcutaneous fat, and perigonadal adipose tissue were harvested from mCMV infected and uninfected age matched controls. Each tissue was collected into a microcentrifuge tube containing 1 mL of Qiazol and autoclaved glass beads, and snap frozen in liquid nitrogen. Following thaw, samples were homogenized by bead beating with two 2-minute cycles. DNA was extracted from each sample per the Qiazol manufacturer’s protocol. qPCR was performed using PowerUP SYBR Green Master Mix on an Applied Biosciences Step One real-time PCR system using the following cycle protocol: an initial step at 2 min 50°C followed by 95° for 10 min, followed by 40 cycles of 95° for 15 sec, 60° for 1 min. Recombinant plasmids containing IE1 and C57/BL6 β-actin were used as template to establish standard curves for quantification. The primer sequences were as follows: IE1-fw (5’- CCC TCT CCT AAC TCT CCC TTT-3’) and IEI-rv (5’-TGG TGC TCT TTT CCC GTG-3’), β-actin-fw (5’-AGC TCA TTG TAG AAG GTG TGG-3’) and β-actin-rv (5’-GGT GGG AAT GGG TCA GAA G-3’). Cycle 32 was set as a negative cut-off based on uninfected controls. Primer sets and recombinant plasmids were gifted by Wayne Yokoyama, MD, Washington University in St. Louis.

### Real-time PCR quantification of viral genome load in FACS-purified cell subsets

8-week-old C57BL/6J female mice were i.p. injected with 10^6^ pfu of bacterial artificial chromosome–derived mCMV (pSM3fr-MCK-2 full-length [73]) and sacrificed at 90d or at greater than 240d p.i. Perigonadal adipose tissue stromal vascular fractions were isolated as described previously [74], stained with antibodies and FACS-sorted into CD45- and CD45+CD11b+ subsets. DNA was extracted using QIAamp DNA Micro Kit (QIAGEN) according to manufacturer’s protocol. Real-time PCR quantification of viral genome load was performed as described previously [75] with modifications. Briefly, equivalent volumes of each DNA sample were analyzed in qPCR reactions with primer pairs specific for either the viral gene M55/gB or the mouse gene Pthrp. Reactions were set up using Fast EvaGreen qPCR master mix (Biotium, Fremont, CA) and run in a LightCycler480 (Roche, Mannheim, Germany) using the following cycling protocol: an initial step of 2 min at 95°C followed by 50 cycles of 10 s at 95°C, 20 s at 56°C, and 30 s at 72°C. Specificity of the amplicons was confirmed through melting curve analysis and by electrophoresis on agarose gels. Absence of cross-contamination was ascertained by parallel assessment of negative water controls and of DNA samples from non-infected animals. A recombinant plasmid standard containing sequences of both gB and Pthrp genes [75] was used as a template to establish standard curves for quantification. The dynamic range of the assay stretched from 101 to106 mCMV genome copies per reaction. The following primer sequences were used: gB-fw (5′- GCAGTCTAGTCGCTTTCTGC-3′) and gB-rev (5′-AAGGCGTGGACTAGCGATAA-3′); Pthrp-fw (5′- GGTATCTGCCCTCATCGTCTG-3′) and Pthrp-rev (5′-CGTTTCTTCCTCCACCATCTG-3′).

### Flow cytometry

Isolated cells were stained using flow cytometry reagents as indicated in Table 1. Dead cells (identified as 7-Amino-Actinomycin D+ or using LIVE/DEAD Fixable Dead Cell Staining Kits) and cell aggregates (identified on FSC-A versus FSC-W scatter plots) were excluded from all analyses. Cells were plated into 96-well round bottom plates (Costar). Cells were treated with FcBlock (anti-CD16/32) in PBS supplemented with 2% BSA (FACs buffer) for 10 minutes at 4 C and then surface staining antibodies, also in FACs buffer, added for an additional 45 minutes at 4 C. In experiments requiring intravascular staining animals were injected with 3 ug of anti-CD45 antibody in 50 ul of PBS retro-orbitally and waiting 5 minutes prior to sacrificing animals. After initial staining steps, cells were washed three times FACs buffer and then stained using LIVE/DEAD viability dye in PBS alone for 30 minutes at 4 C. Finally, cells were washed once with PBS and three times with FACs. Cells were fixed in BD Cytofix following manufacturers protocol and then washed three times prior to analysis. Data acquisition was performed on a custom-made, four-laser BD Fortessa flow cytometer (Becton Dickinson), and was analyzed using FlowJo software (Tree Star). Cell sorting was performed on a FACSAriaII (BD Biosciences). Gating was informed by using fluorescence minus one (FMO) controls.

**Table 1:** Key resources table.

Reagent	Source	Identifier
Adiponectin Mouse ELISA Kit	ThermoFisher Scientific	KMP0041
anti-mouse CD103e	BioLegend	121419
anti-mouse CD127	BioLegend	135035
anti-mouse CD3	BioLegend	100229
anti-mouse CD4	BioLegend	100541
anti-mouse CD44	BioLegend	103011
anti-mouse CD62L	ThermoFisher Scientific	RM4317
anti-mouse CD69	BioLegend	104507
anti-mouse CD8	BioLegend	100733
anti-mouse KLRG1	BioLegend	138415
anti-mouse CD3	BioLegend	100216
anti-mouse CD45.2	BioLegend	109822
anti-mouse CD45.2	BioLegend	109807
anti-mouse CD11b	BioLegend	101216
Purified anti-mouse CD16/32	BioLegend	101301
LEGENDplex Mouse Inflammation Panel	BioLegend	740446
Collagenase D	Sigma-Aldrich	11088858001
RNeasy Lipid Tissue Mini Kit	Qiagen	74804
QIAzol Lysis Reagent	Qiagen	79306
RT^2^ Profiler PCR Array Mouse Insulin Resistance	Qiagen	330231
H-2D(b) MCMV M45 985–993 HGIRNASFI	NIH Tetramer Core Facility	41184
H-2K(b) MCMV m139 419–426 TVYGFCLL	NIH Tetramer Core Facility	41186
H-2K(b) MCMV m38 316–323 SSPPMFRV	NIH Tetramer Core Facility	41185
LIVE/DEAD Fixable Aqua Dead Cell Kit	ThermoFisher Scientific	L34957
LIVE/DEAD Fixable Near-IR Dead Cell Kit	ThermoFisher Scientific	L10119
7-AAD Viability Staining Solution	BioLegend	420404
Mouse Inflammation Panel LegendPlex	BioLegend	740150
NP-40 Surfact-Amps Detergent Solution	ThermoFisher Scientific	28324
Protease Inhibitor Cocktail	Sigma-Aldrich	P8340
Mouse Leptin ELISA Kit	Sigma-Aldrich	RAB0334
Insulin Mouse ELISA Kit	ThermoFisher Scientific	EMINS
BD Cytofix/Cytoperm Fixation and Permeabilization Solution	Fisher Scientific	BDB554655
Humalog Insulin (100 U / mL)	Eli Lilly	Vet Prescribed
D-(+) Glucose	Sigma-Aldrich	G8270-25KG
Sodium Pyruvate	Lonza	13-115E

### Collection of adipose tissue homogenate for ELISA and BioLegend LegendPlex

Total epididymal adipose tissue was excised from infected and control animals and weighed to normalize downstream analysis per gram. Adipose was collected into 0.5% NP-40 buffer in PBS plus 1/100 protease inhibitor cocktail and homogenized using Qiagen TissueRuptor. Samples were incubated at room temperature for 30 minutes and then centrifuged at 4700 RPM for 30 minutes at 4 C. Liquid interphase was taken for downstream analysis. Adiponectin and Leptin ELISAs and BioLegend LegendPlex 13-plex Inflammation Panel analyses were carried out following manufacturer protocols.

### Blood glucose and plasma insulin measurements, glucose tolerance test (GTT), insulin tolerance test (ITT), and pyruvate tolerance test (PTT)

Prior to collection of fasted blood glucose and plasma for insulin measurements mice were fasted for at least seven hours. Blood glucose was measured by tail nick and using Bayer Counter Next EZ Glucose Meter. After glucose measurements blood was obtained via retro-orbital bleeding into EDTA treated tubes (ThermoFisher) by centrifugation for 15 minutes in 4 C at 2,000 x g. Insulin was measured using Insulin Mouse ELISA kit (ThermoFisher). HOMA-IR was calculated by multiplying fasted plasma insulin and fasted blood glucose and dividing the product by 22.5, the inverse of this result was taken to represent Insulin Sensitivity [76]. For GTT, ITT, or PTT, following fasting mice were i.p. challenged with 1 mg/kg glucose, or 1 U/kg Humalog insulin (Eli Lilly), or 2.5 g/kg sodium pyruvate in PBS respectively and blood glucose measured as described above.

### Statistical analysis

Statistics were performed in Prism 7.0 (GraphPad Software, La Jolla, CA, USA). Two-tailed Mann Whitney *U* tests with equal SD were carried out on all analyses unless otherwise noted. Significance is noted as follows throughout: ns = not significant, ****P < 0.0001, ***P < 0.001, **P < 0.01, *P < 0.05. All error bars shown are SEM. In all cases, a bar overlies groups compared for significance and the stars as described above denote significance.

## Supporting information

S1 Fig mCMV DNA burden across tissues from d3 and d7 p.i. 12-week-old C57BL/6J mice were infected with 10^5^ pfu of mCMV by the i.p. route and sacrificed at 3d and 7d p.i.. Tissues were snap frozen in Qiazol then DNA and RNA extracted. (A) Total mCMV RNA burden in visceral adipose, spleen, and livers at 3d p.i. (B) Total mCMV DNA burden in subcutaneous adipose, visceral adipose, spleens, and liver was normalized to β-actin. Uninfected animals were used to establish C_T_ cut off at 32. Technical duplicates were run for both RNA and DNA. Data is representative of three independent experiments. n = 3 to 6 total animals per group. Kruskal-Wallis with Dunn’s multiple comparisons. Error bars represent mean ± SEM. *p < 0.05; **p < 0.01; ***p < 0.001; **** p ≤ 0.0001.
(TIF)

S2 Fig Adipose tissue NK cell and macrophage response during infection early, acute, and lifelong infection. 12-week-old C57BL/6J mice were infected with 10^5^ pfu of mCMV by the i.p. route and sacrificed at 3d, 7d and > 450d p.i. Stromal vascular fraction was analyzed by flow cytometry and cell populations quantified (A) NK cells. (B) F480+CD11b+ Macrophages. (C) F480+CD11b+CD11c+ M1 Macrophages. (D) F480+CD11b+CD206+ M2 Macrophages. Data are pooled data of two independent experiments. n = 4–9 mice per group. Error bars represent mean ± SEM. Lifelong and aged matched control groups were analyzed by unpaired two-tailed Mann-Whitney U test. Control, 3 dpi, and 7 dpi were analyzed by Kruskal-Wallis with Dunn’s multiple comparisons. *p < 0.05; **p < 0.01; ***p < 0.001; **** p ≤ 0.0001.
(TIF)

S3 Fig Acute mCMV infection alters adipose cytokine milieu. 12-week-old C57BL/6J mice were infected with 10^5^ pfu of mCMV by the i.p. route and sacrificed at 7d p.i.. Total adipose tissue was homogenized and analyzed by BioLegend LegendPlex for (A) IFNγ; (B) CCL2. Data are pooled results of two independent experiments. n = 10 uninfected and 10 infected animals total. Error bars represent mean ± SEM. *p < 0.05; **p < 0.01; ***p < 0.001; **** p ≤ 0.0001 by unpaired two-tailed Mann-Whitney U test.
(TIF)

S4 Fig Inflammatory transcripts are upregulated at 7d p.i.. 12-week-old C57BL/6J mice were infected with 10^5^ pfu of mCMV by the i.p. route and sacrificed at 7d p.i.. Transcriptome was analyzed using RT2 Insulin Resistance Miniarray Profiler and presented as a volcano plot. All housekeeping genes were used for normalization. A total of 3 infected and 3 uninfected animals were used. A cut off of 35 cycles was set as undetectable per manufacturer’s suggestions.
(TIF)

S5 Fig Acute mCMV infection alters adipose adipokine milieu. 12-week-old C57BL/6J mice were infected with 10^5^ pfu of mCMV by the i.p. route and sacrificed at 7d p.i.. Total adipose tissue was homogenized and analyzed by ELISA for (A) Adiponectin; and (B) Leptin. Data are pooled results of two independent experiments. n = 5 uninfected and 8 infected animals total. Error bars represent mean ± SEM. *p < 0.05; **p < 0.01; ***p < 0.001; **** p ≤ 0.0001 by unpaired two-tailed Mann-Whitney U test.
(TIF)

S6 Fig Longitudinal fat pad weight change and body weight of lifelong infected animals and aged-matched controls. 12-week-old C57BL/6J mice were infected with 10^5^ pfu of mCMV by the i.p. route. At sacrifice times as noted through the manuscript, adipose tissue was collected and analyzed. (A) Total weight of epididymal fat pad at time of harvest. (B) Body weight of mice infected for greater than 450 days and their aged matched counterparts. Data is pooled from multiple experiments. n = 5–35 total animals per group.
(TIF)

S7 Fig mCMV burden in CD45- and CD45+ adipose tissue cells. 8-week-old C57BL/6J female mice were i.p. injected with 10^6^ pfu of bacterial artificial chromosome–derived mCMV (pSM3fr-MCK-2 full-length and sacrificed at 90d or at greater than 240d p.i. Perigonadal adipose tissue stromal vascular fractions were isolated and stained with antibodies and FACS-sorted into CD45- and CD45+CD11b+ subsets. (A) FAC-sort purity (B) mCMV DNA burden in CD45- vs CD45+CD11b+ subsets of visceral adipose tissue of 10 months post infected mice.
(TIF)

S8 Fig Dual expression of CD69 and CD103e is not significantly different between CD8 T cells in lifelong mCMV infected and uninfected adipose tissue. 12-week-old C57BL/6J mice were infected with 10^5^ pfu of mCMV by the i.p. route. At greater than 450d p.i. mice were sacrificed Stromal vascular fraction was analyzed by flow cytometry and cell populations quantified. Dual expression of CD69+CD103e+ CD44+ CD8 T cells were quantified. Data are pooled results of two individual experiments. n = 10 infected and n = 10 uninfected. Error bars represent mean ± SEM. *p < 0.05; **p < 0.01; ***p < 0.001; **** p ≤ 0.0001 by unpaired two-tailed Mann-Whitney U test within genotypes.
(TIF)

S9 Fig B2m is required for manifestation of hyperglycemia during lifelong mCMV infection. 12-week-old C57BL/6J and B2m KO mice were infected with 10^5^ pfu of mCMV by the i.p. route. After greater than 300d p.i. mice were fasted for 6 hours and fasted blood glucose was analyzed. Data are representative of two individual experiments. n = 10 infected B2m and n = 9 uninfected B2m. n = 5 infected C57BL/6 and n = 4 uninfected C57BL/6. Error bars represent mean ± SEM. *p < 0.05; **p < 0.01; ***p < 0.001; **** p ≤ 0.0001 by unpaired two-tailed Mann-Whitney U test within genotypes.
(TIF)

S10 Fig Systemic insulin sensitivity and gluconeogenesis are not altered in lifelong mCMV infected animals. 12-week-old C57BL/6J mice were infected with 10^5^ pfu of mCMV by the i.p. route. After greater than 450d p.i. mice were challenged with insulin tolerance (ITT) and pyruvate tolerance tests (PTT). (A) Percent change of fasted blood glucose compared to Time 0 after i.p. injection of 1 U / kg insulin. (B) Percent change of fasted blood glucose compared to Time 0 after i.p. injection of 2 mg / kg sodium pyruvate. Data are representative of two repeated experiments for each test. n = 18 infected and 10 uninfected animals in total.
(TIF)

S11 Fig Lifelong mCMV infection does not alter adiponectin levels. 12-week-old C57BL/6J mice were infected with 10^5^ pfu of mCMV by the i.p. route and sacrificed at >450d p.i.. Total adipose tissue was homogenized and analyzed by ELISA for Adiponectin. Data are pooled results of two independent experiments. n = 12 uninfected and 17 infected animals total. Error bars represent mean ± SEM. *p < 0.05; **p < 0.01; ***p < 0.001; **** p ≤ 0.0001 by unpaired two-tailed Mann-Whitney U test.
(TIF)
