# Supplementary figures and images for: Life-long control of cytomegalovirus (CMV) by T resident memory cells in the adipose tissue results in inflammation and hyperglycemia
Source: PLoS Pathog. 2019 Jun 20;15(6):e1007890. doi: 10.1371/journal.ppat.1007890 (PMC6605679; doi:10.1371/journal.ppat.1007890)

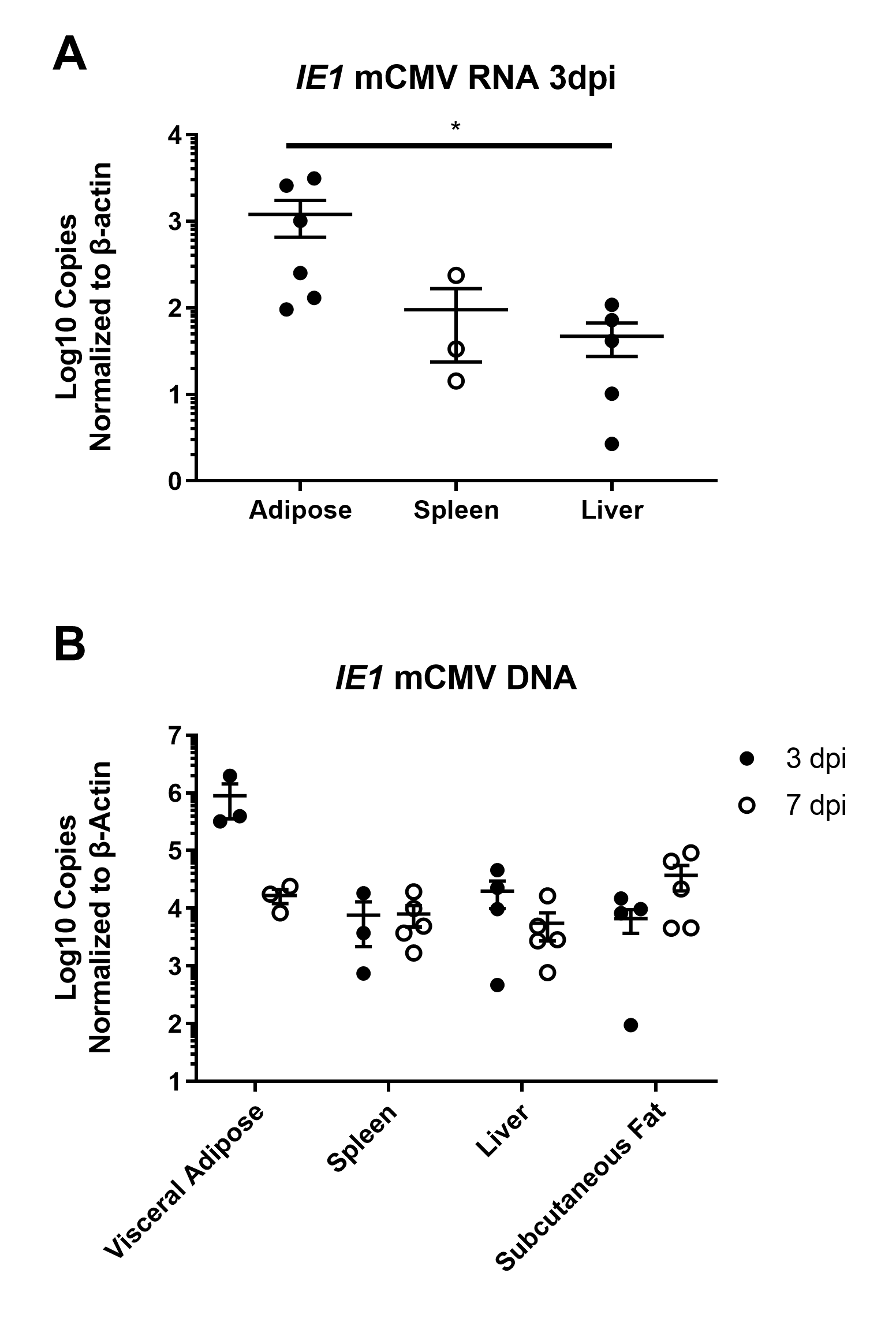

Supplement: S1 Fig — 12-week-old C57BL/6J mice were infected with 105 pfu of mCMV by the i.p. route and sacrificed at 3d and 7d p.i.. Tissues were snap frozen in Qiazol then DNA and RNA extracted. (A) Total mCMV RNA burden in visceral adipose, spleen, and livers at 3d p.i. (B) Total mCMV DNA burden in subcutaneous adipose, visceral adipose, spleens, and liver was normalized to β-actin. Uninfected animals were used to establish CT cut off at 32. Technical duplicates were run for both RNA and DNA. Data is representative of three independent experiments. n = 3 to 6 total animals per group. Kruskal-Wallis with Dunn’s multiple comparisons. Error bars represent mean ± SEM. *p < 0.05; **p < 0.01; ***p < 0.001; **** p ≤ 0.0001. (TIF) [file ppat.1007890.s001.tif]

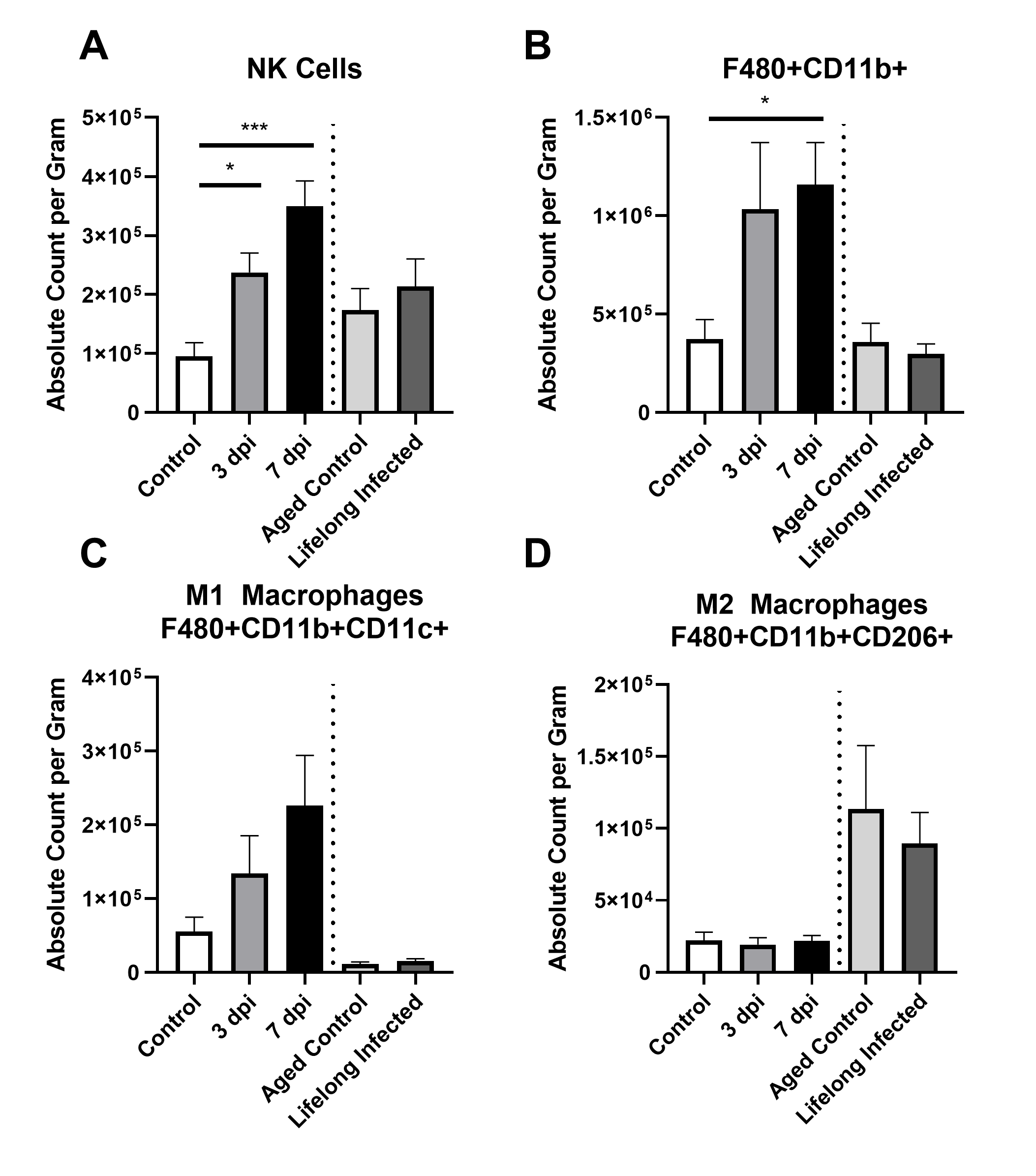

Supplement: S2 Fig — 12-week-old C57BL/6J mice were infected with 105 pfu of mCMV by the i.p. route and sacrificed at 3d, 7d and > 450d p.i. Stromal vascular fraction was analyzed by flow cytometry and cell populations quantified (A) NK cells. (B) F480+CD11b+ Macrophages. (C) F480+CD11b+CD11c+ M1 Macrophages. (D) F480+CD11b+CD206+ M2 Macrophages. Data are pooled data of two independent experiments. n = 4–9 mice per group. Error bars represent mean ± SEM. Lifelong and aged matched control groups were analyzed by unpaired two-tailed Mann-Whitney U test. Control, 3 dpi, and 7 dpi were analyzed by Kruskal-Wallis with Dunn’s multiple comparisons. *p < 0.05; **p < 0.01; ***p < 0.001; **** p ≤ 0.0001. (TIF) [file ppat.1007890.s002.tif]

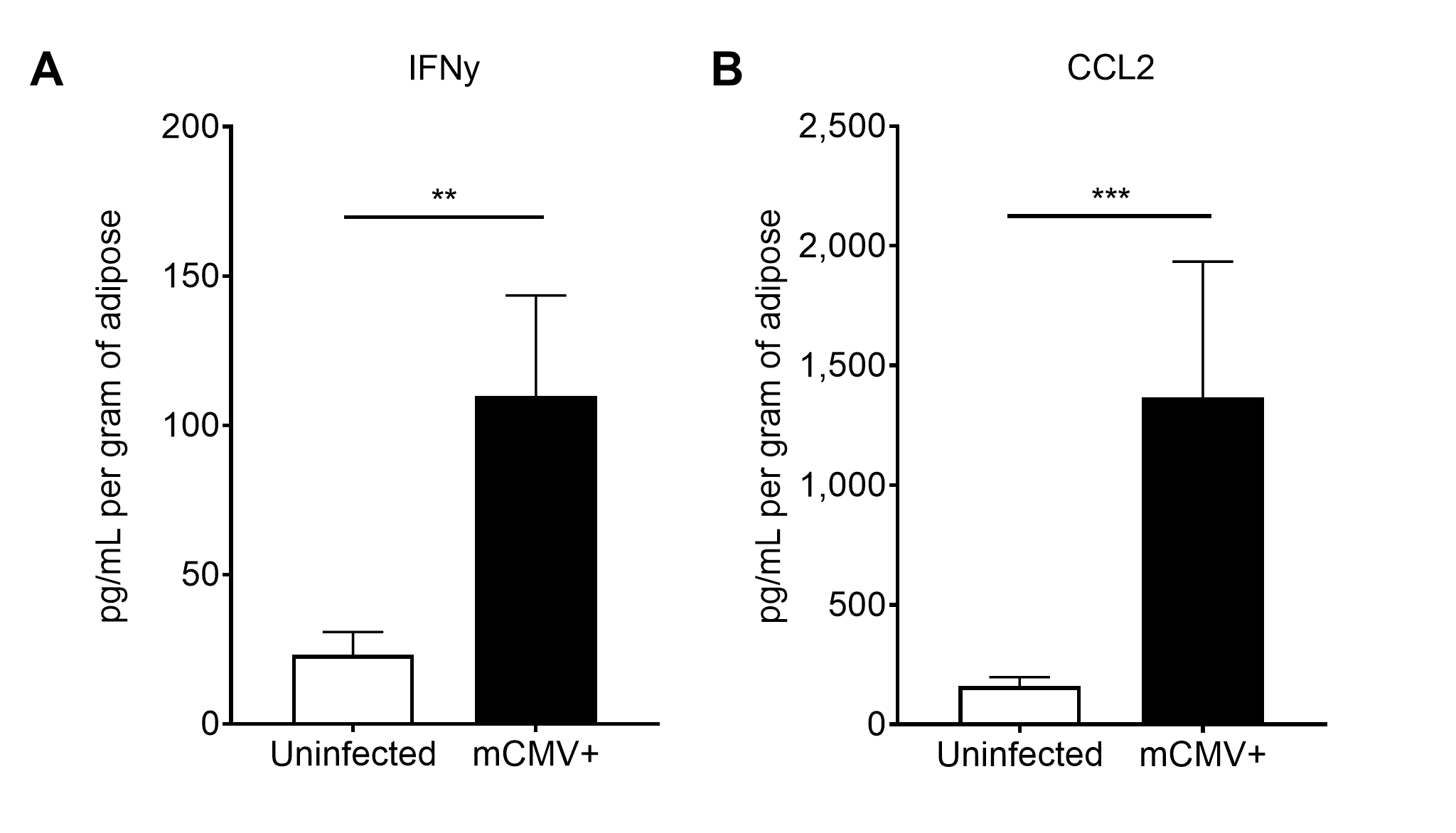

Supplement: S3 Fig — 12-week-old C57BL/6J mice were infected with 105 pfu of mCMV by the i.p. route and sacrificed at 7d p.i.. Total adipose tissue was homogenized and analyzed by BioLegend LegendPlex for (A) IFNγ; (B) CCL2. Data are pooled results of two independent experiments. n = 10 uninfected and 10 infected animals total. Error bars represent mean ± SEM. *p < 0.05; **p < 0.01; ***p < 0.001; **** p ≤ 0.0001 by unpaired two-tailed Mann-Whitney U test. (TIF) [file ppat.1007890.s003.tif]

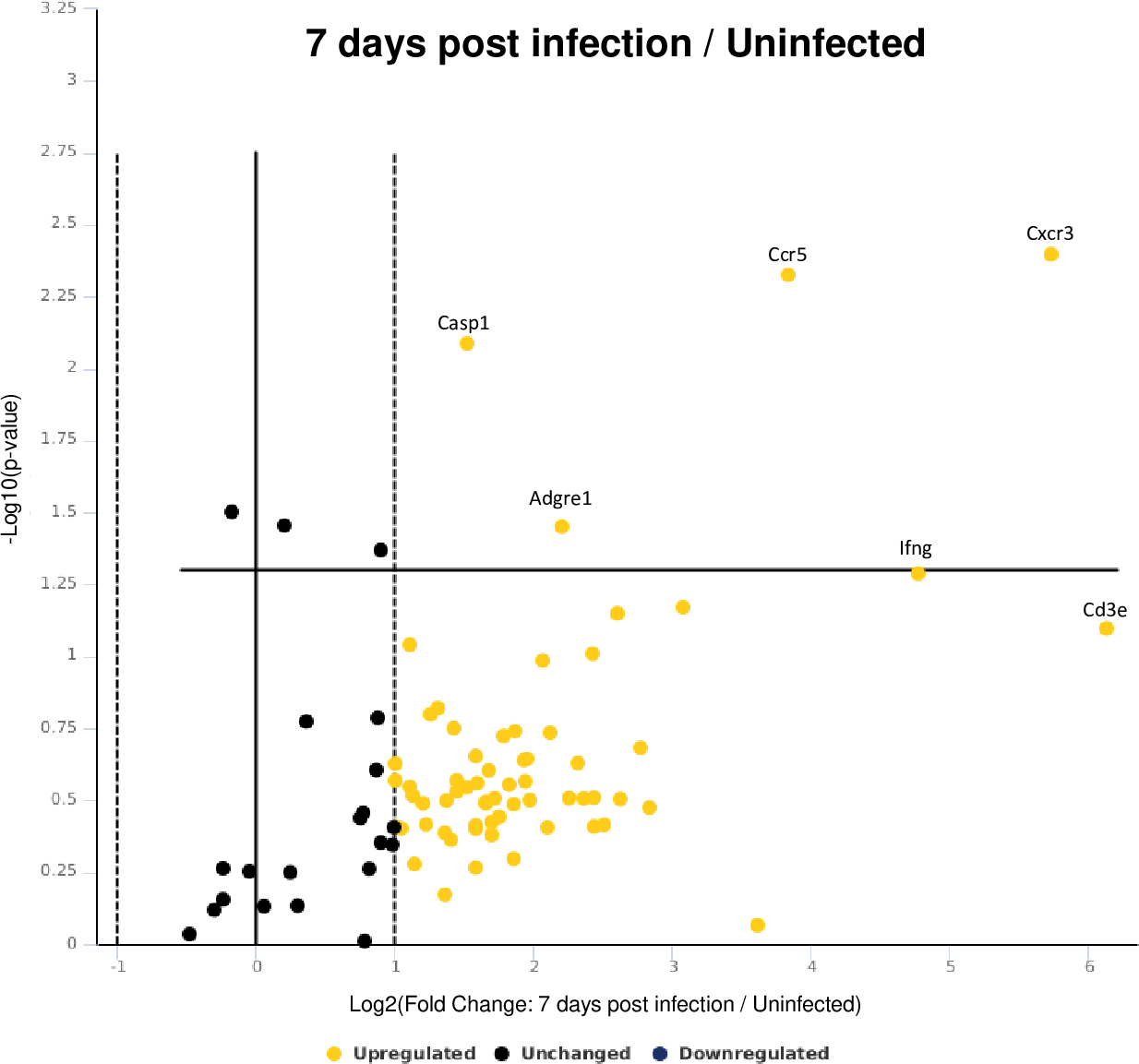

Supplement: S4 Fig — 12-week-old C57BL/6J mice were infected with 105 pfu of mCMV by the i.p. route and sacrificed at 7d p.i.. Transcriptome was analyzed using RT2 Insulin Resistance Miniarray Profiler and presented as a volcano plot. All housekeeping genes were used for normalization. A total of 3 infected and 3 uninfected animals were used. A cut off of 35 cycles was set as undetectable per manufacturer’s suggestions. (TIF) [file ppat.1007890.s004.tif]

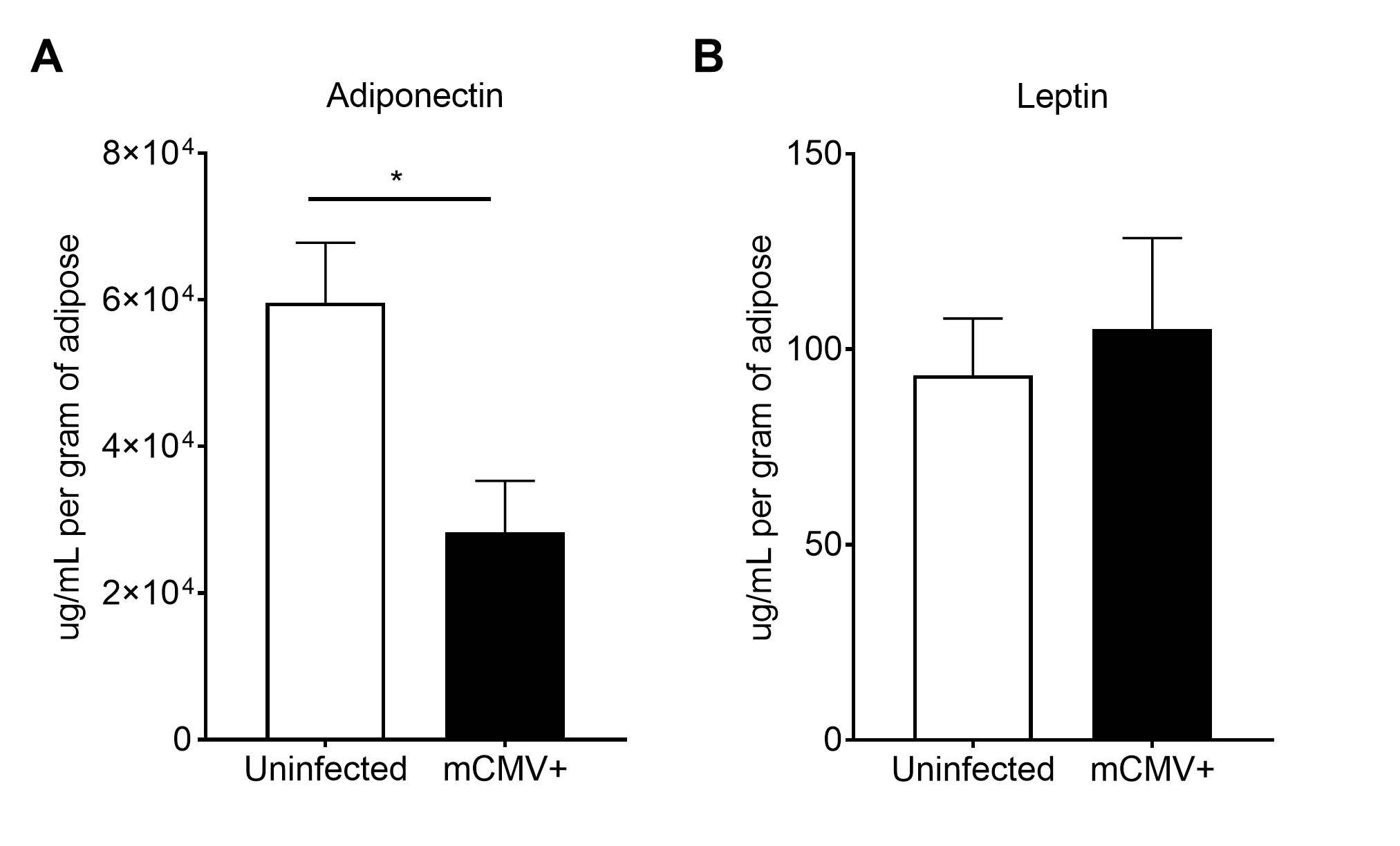

Supplement: S5 Fig — 12-week-old C57BL/6J mice were infected with 105 pfu of mCMV by the i.p. route and sacrificed at 7d p.i.. Total adipose tissue was homogenized and analyzed by ELISA for (A) Adiponectin; and (B) Leptin. Data are pooled results of two independent experiments. n = 5 uninfected and 8 infected animals total. Error bars represent mean ± SEM. *p < 0.05; **p < 0.01; ***p < 0.001; **** p ≤ 0.0001 by unpaired two-tailed Mann-Whitney U test. (TIF) [file ppat.1007890.s005.tif]

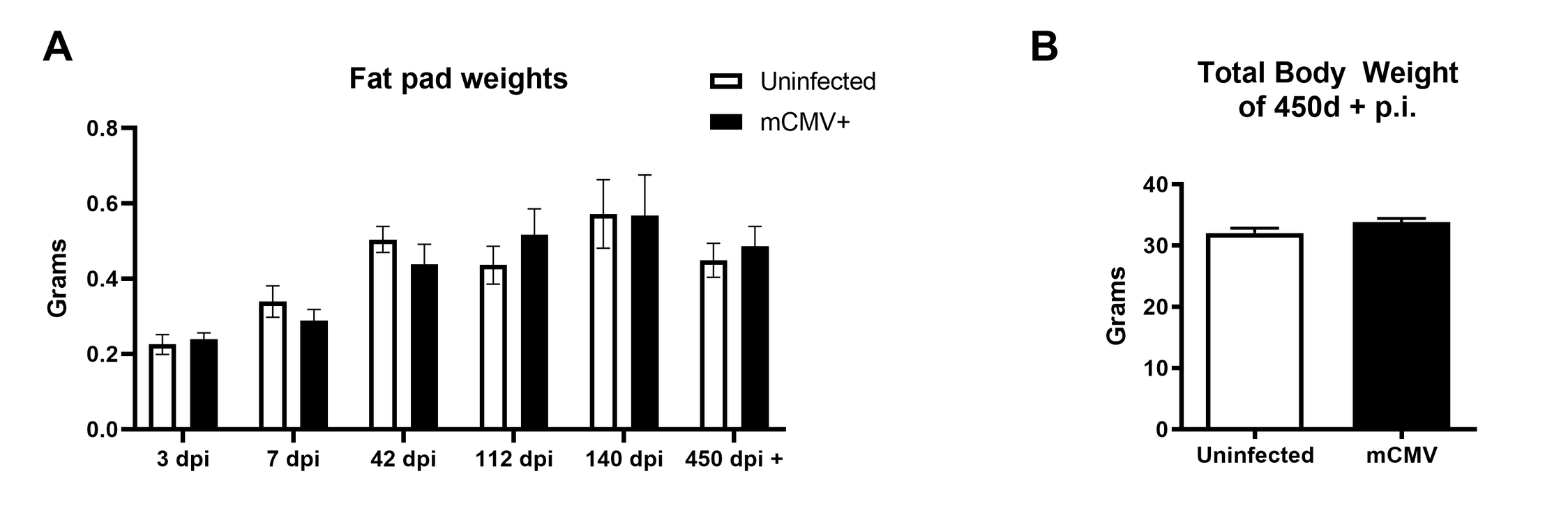

Supplement: S6 Fig — 12-week-old C57BL/6J mice were infected with 105 pfu of mCMV by the i.p. route. At sacrifice times as noted through the manuscript, adipose tissue was collected and analyzed. (A) Total weight of epididymal fat pad at time of harvest. (B) Body weight of mice infected for greater than 450 days and their aged matched counterparts. Data is pooled from multiple experiments. n = 5–35 total animals per group. (TIF) [file ppat.1007890.s006.tif]

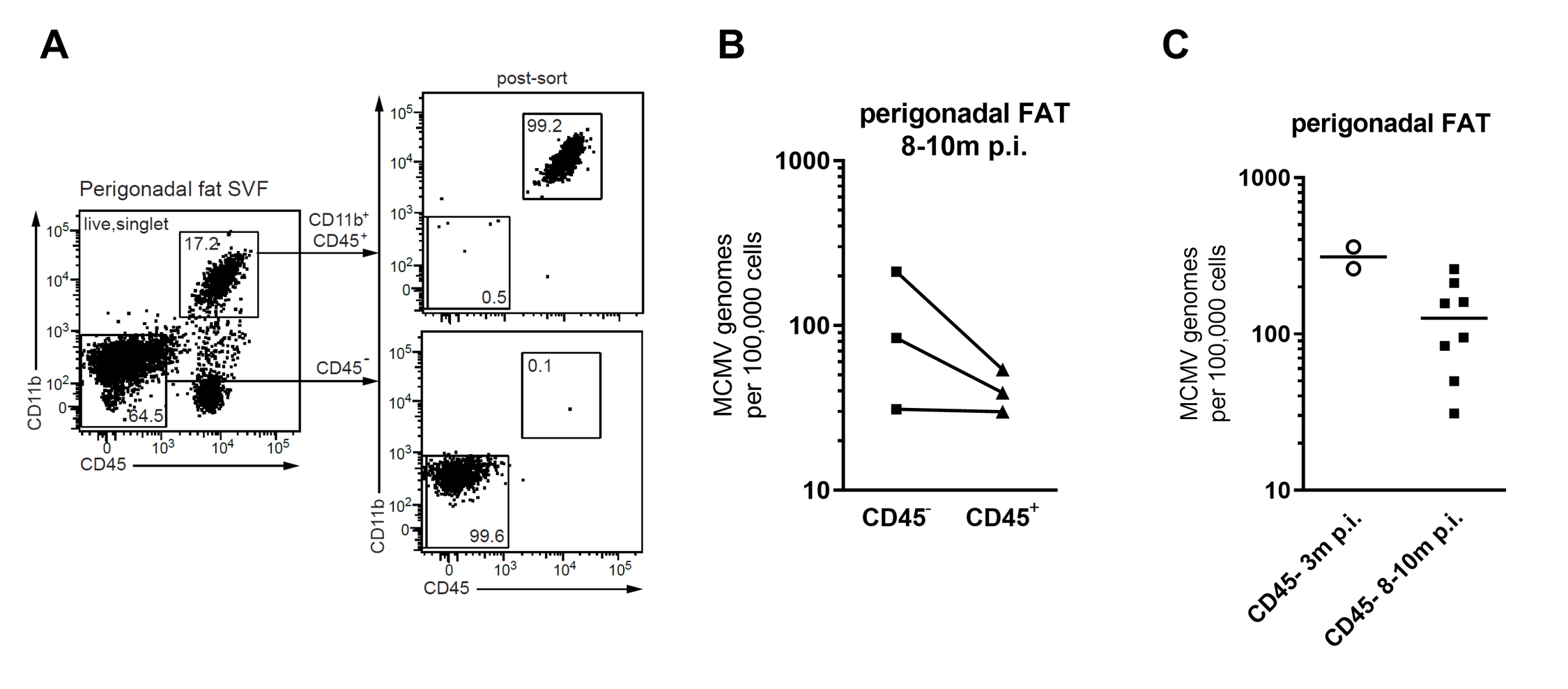

Supplement: S7 Fig — 8-week-old C57BL/6J female mice were i.p. injected with 106 pfu of bacterial artificial chromosome–derived mCMV (pSM3fr-MCK-2 full-length and sacrificed at 90d or at greater than 240d p.i. Perigonadal adipose tissue stromal vascular fractions were isolated and stained with antibodies and FACS-sorted into CD45- and CD45+CD11b+ subsets. (A) FAC-sort purity (B) mCMV DNA burden in CD45- vs CD45+CD11b+ subsets of visceral adipose tissue of 10 months post infected mice. (TIF) [file ppat.1007890.s007.tif]

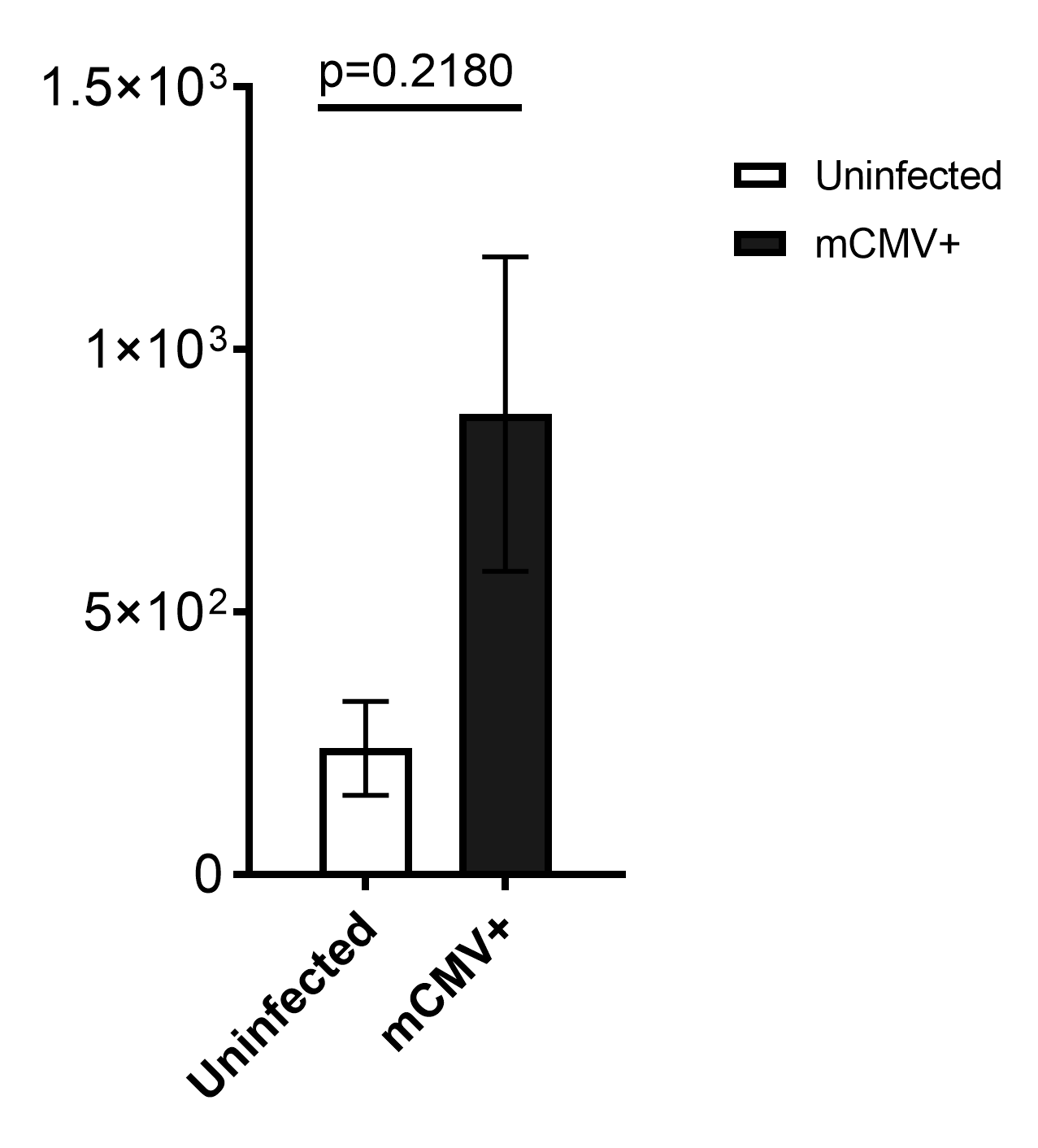

Supplement: S8 Fig — 12-week-old C57BL/6J mice were infected with 105 pfu of mCMV by the i.p. route. At greater than 450d p.i. mice were sacrificed Stromal vascular fraction was analyzed by flow cytometry and cell populations quantified. Dual expression of CD69+CD103e+ CD44+ CD8 T cells were quantified. Data are pooled results of two individual experiments. n = 10 infected and n = 10 uninfected. Error bars represent mean ± SEM. *p < 0.05; **p < 0.01; ***p < 0.001; **** p ≤ 0.0001 by unpaired two-tailed Mann-Whitney U test within genotypes. (TIF) [file ppat.1007890.s008.tif]

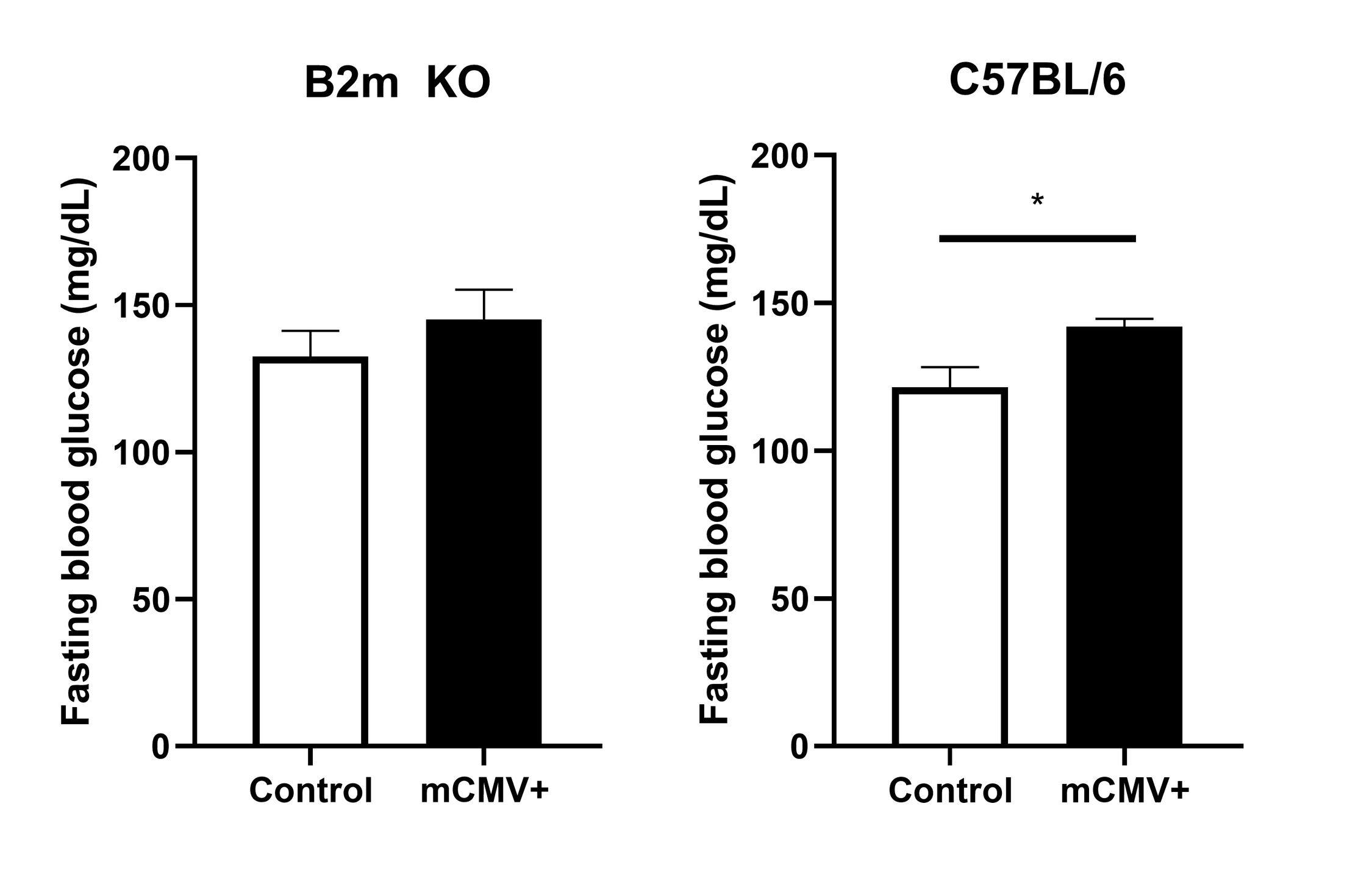

Supplement: S9 Fig — 12-week-old C57BL/6J and B2m KO mice were infected with 105 pfu of mCMV by the i.p. route. After greater than 300d p.i. mice were fasted for 6 hours and fasted blood glucose was analyzed. Data are representative of two individual experiments. n = 10 infected B2m and n = 9 uninfected B2m. n = 5 infected C57BL/6 and n = 4 uninfected C57BL/6. Error bars represent mean ± SEM. *p < 0.05; **p < 0.01; ***p < 0.001; **** p ≤ 0.0001 by unpaired two-tailed Mann-Whitney U test within genotypes. (TIF) [file ppat.1007890.s009.tif]

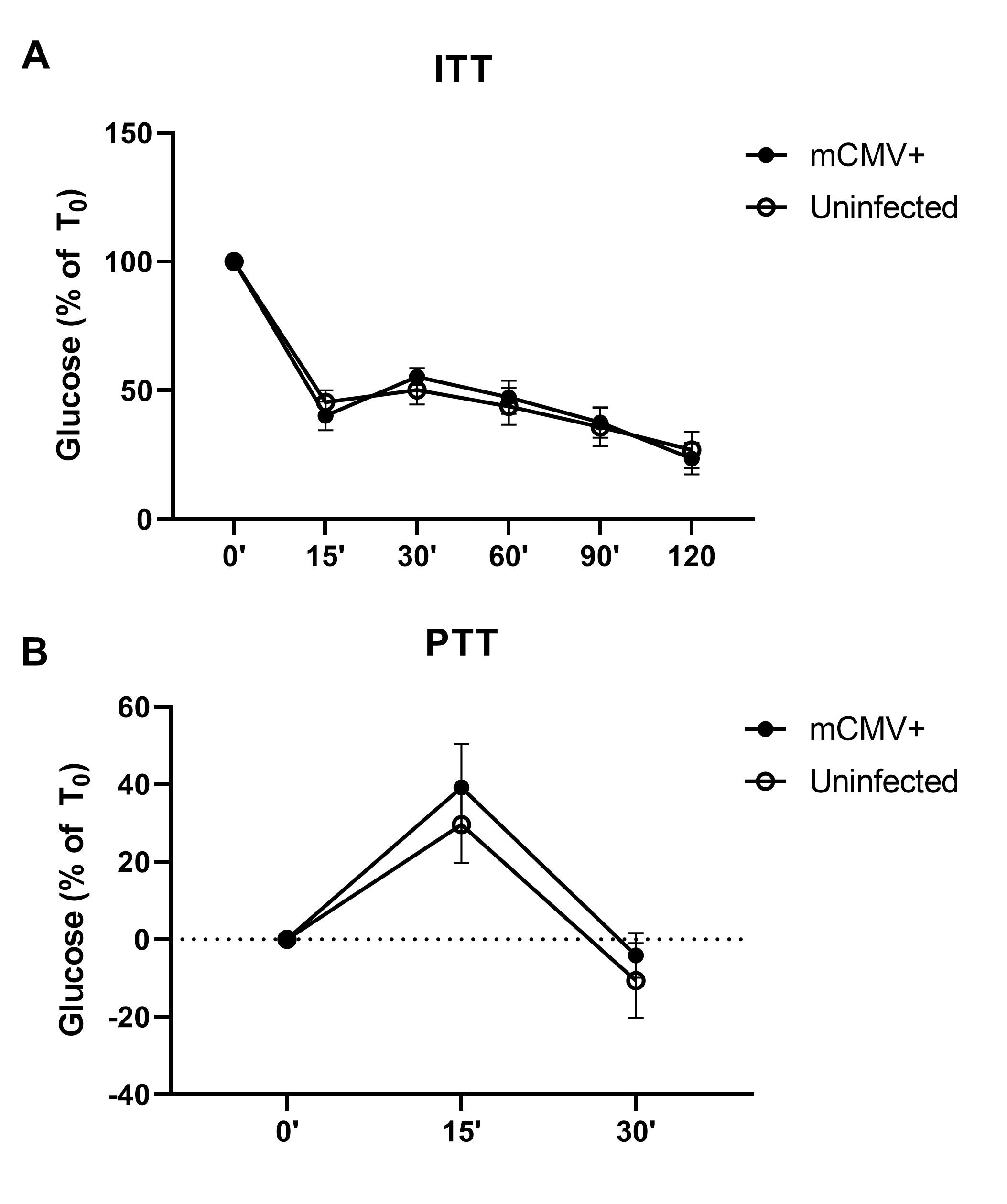

Supplement: S10 Fig — 12-week-old C57BL/6J mice were infected with 105 pfu of mCMV by the i.p. route. After greater than 450d p.i. mice were challenged with insulin tolerance (ITT) and pyruvate tolerance tests (PTT). (A) Percent change of fasted blood glucose compared to Time 0 after i.p. injection of 1 U / kg insulin. (B) Percent change of fasted blood glucose compared to Time 0 after i.p. injection of 2 mg / kg sodium pyruvate. Data are representative of two repeated experiments for each test. n = 18 infected and 10 uninfected animals in total. (TIF) [file ppat.1007890.s010.tif]

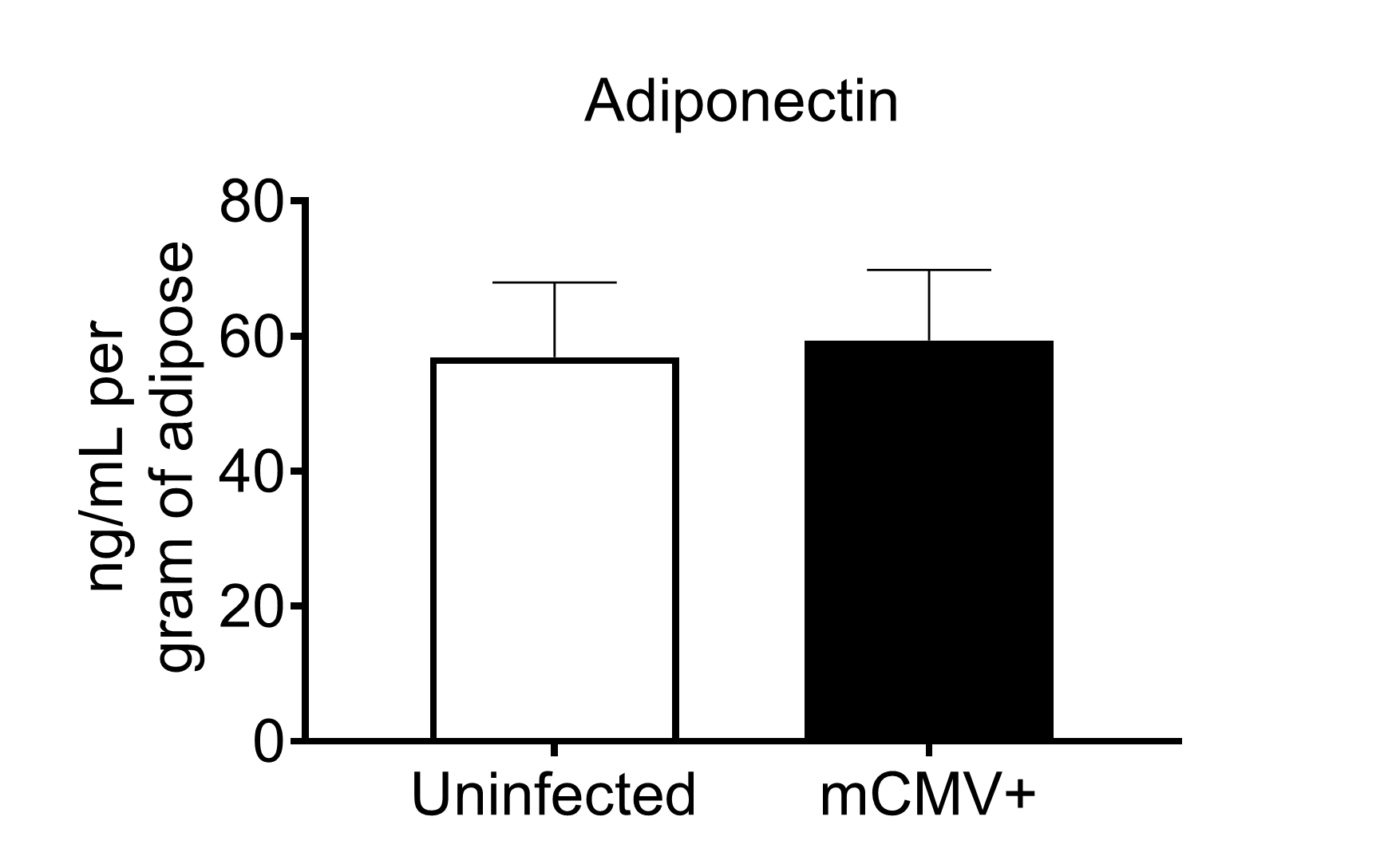

Supplement: S11 Fig — 12-week-old C57BL/6J mice were infected with 105 pfu of mCMV by the i.p. route and sacrificed at >450d p.i.. Total adipose tissue was homogenized and analyzed by ELISA for Adiponectin. Data are pooled results of two independent experiments. n = 12 uninfected and 17 infected animals total. Error bars represent mean ± SEM. *p < 0.05; **p < 0.01; ***p < 0.001; **** p ≤ 0.0001 by unpaired two-tailed Mann-Whitney U test. (TIF) [file ppat.1007890.s011.tif]
